# Supplementary material for: DC-STAMP activates the PI3K/AKT/mTOR signaling pathway to regulate PANoptosis in acute myeloid leukemia
Source: PLoS One. 2026 Jan 16;21(1):e0339670. doi: 10.1371/journal.pone.0339670 (PMC12810915; doi:10.1371/journal.pone.0339670)
Supplement: S1 File — (PDF) [file pone.0339670.s001.pdf]

Fig2C

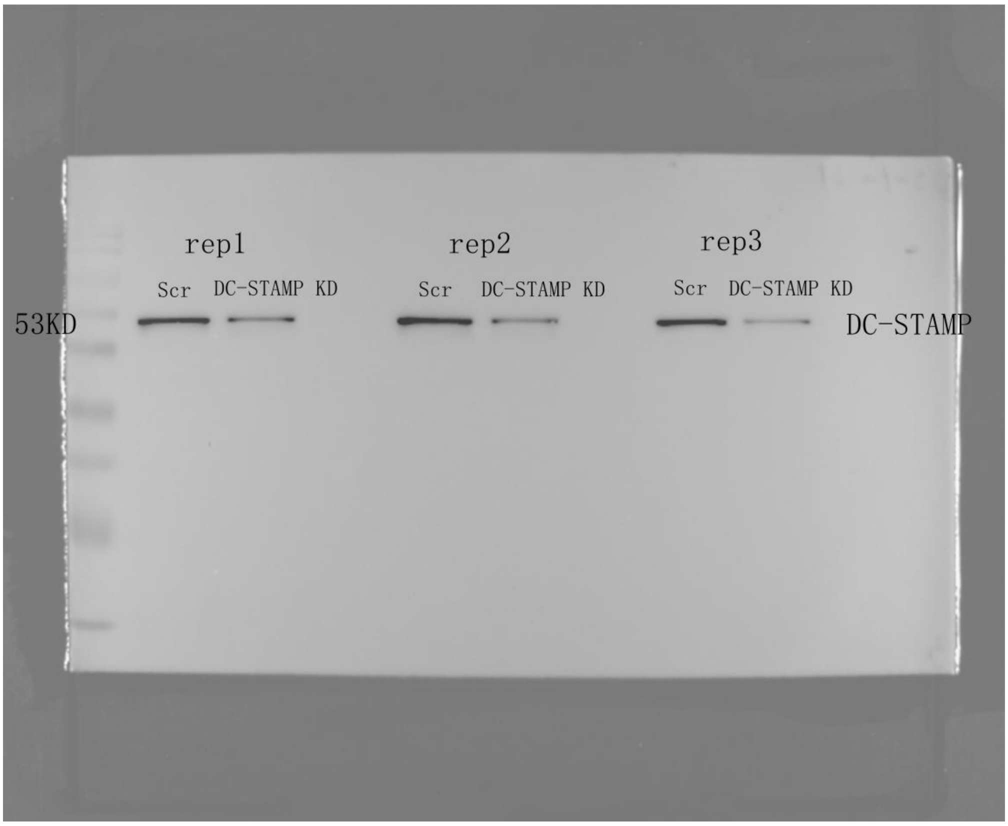
rep1
rep2
rep3
Scr
DC-STAMP KD
Scr
DC-STAMP KD
Scr
DC-STAMP KD
53KD
DC-STAMP

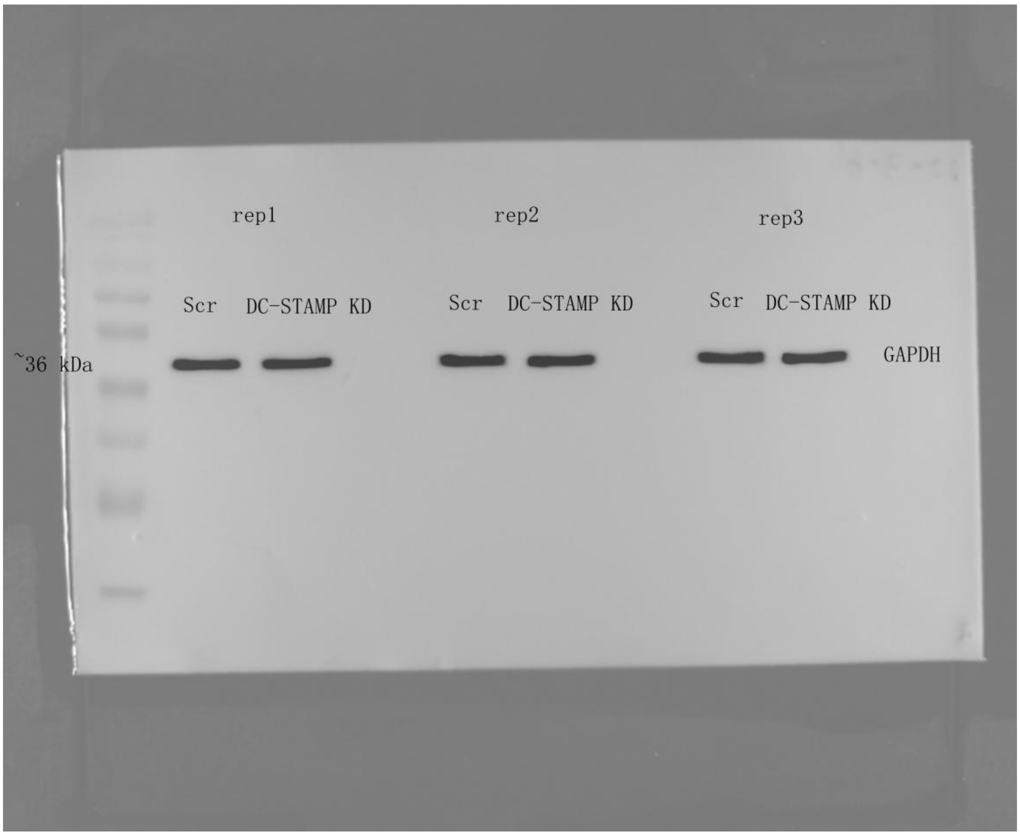
rep1
rep2
rep3
Scr
DC-STAMP KD
Scr
DC-STAMP KD
Scr
DC-STAMP KD
~36 kDa
GAPDH

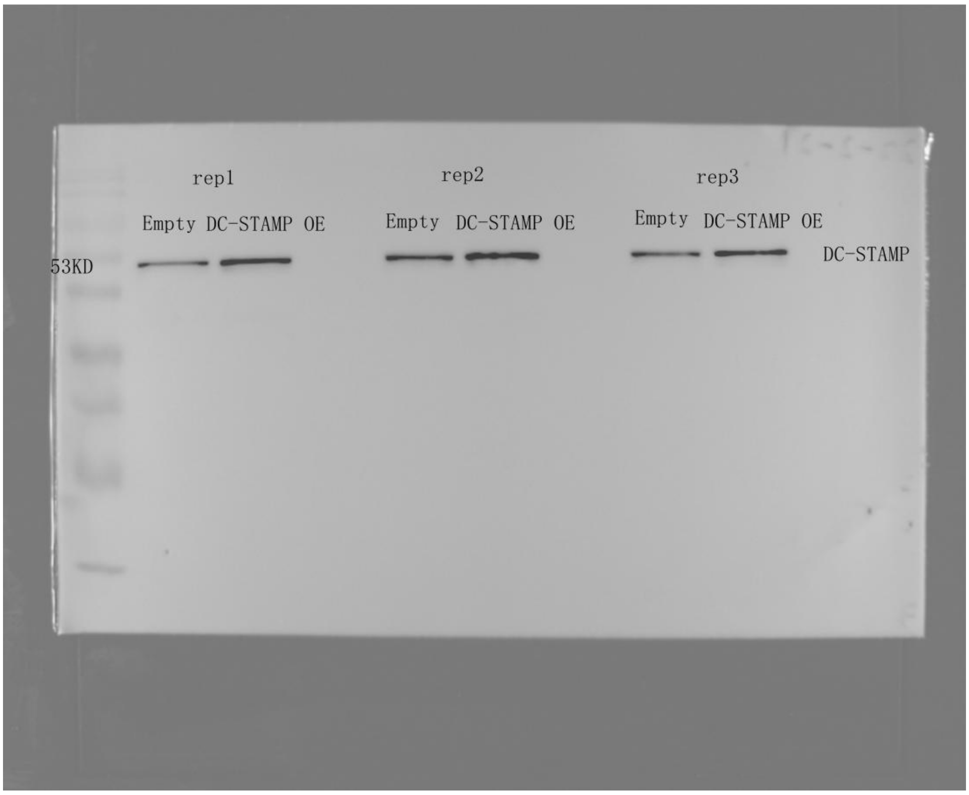
rep1
rep2
rep3
Empty DC-STAMP OE
Empty DC-STAMP OE
Empty DC-STAMP OE
53KD
DC-STAMP

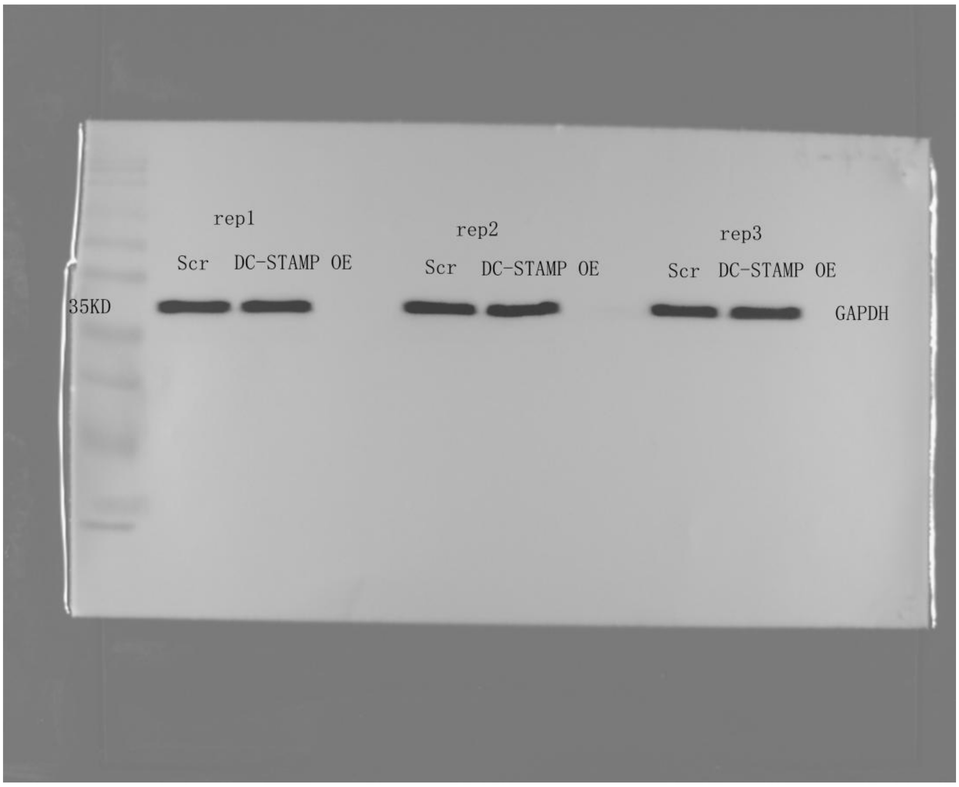
rep1
rep2
rep3
Scr DC-STAMP OE
Scr DC-STAMP OE
Scr DC-STAMP OE
35KD
GAPDH

Fig4B

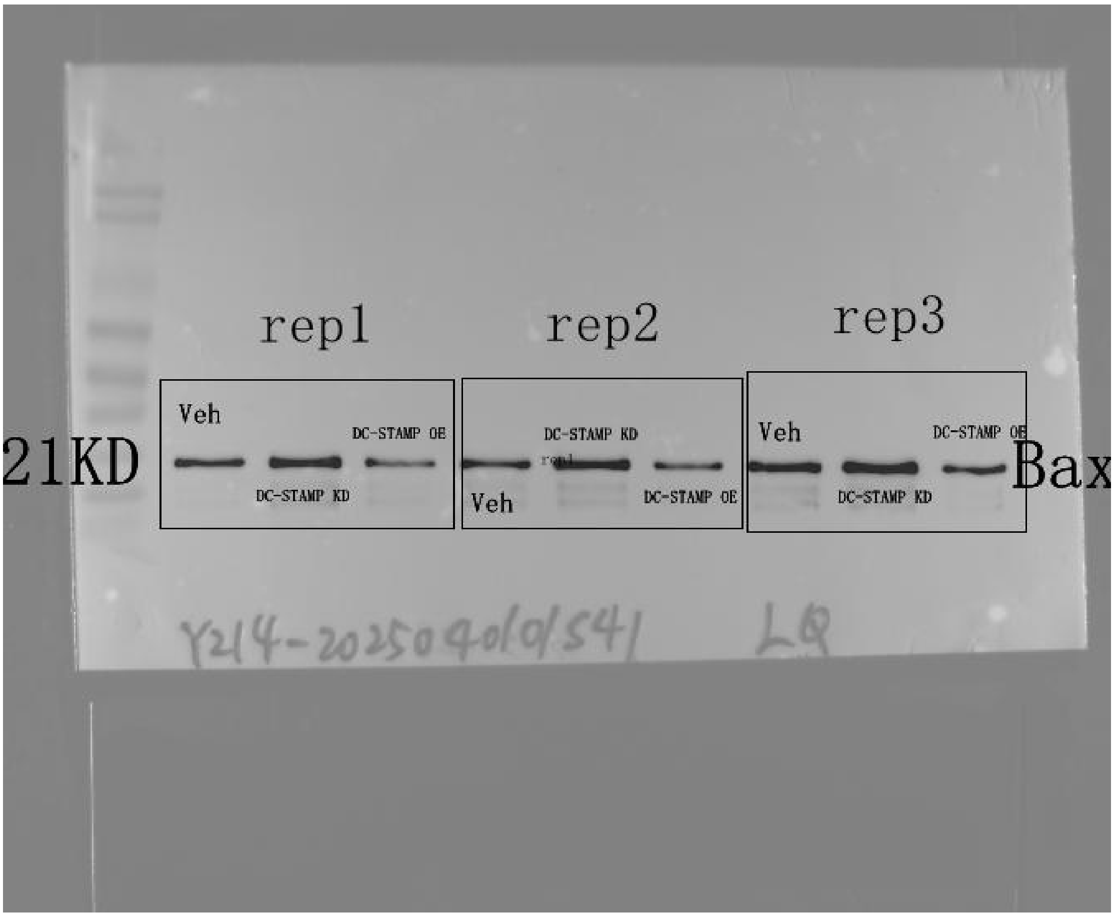
rep1
rep2
rep3
21KD
Veh
DC-STAMP KD
DC-STAMP OE
Bax

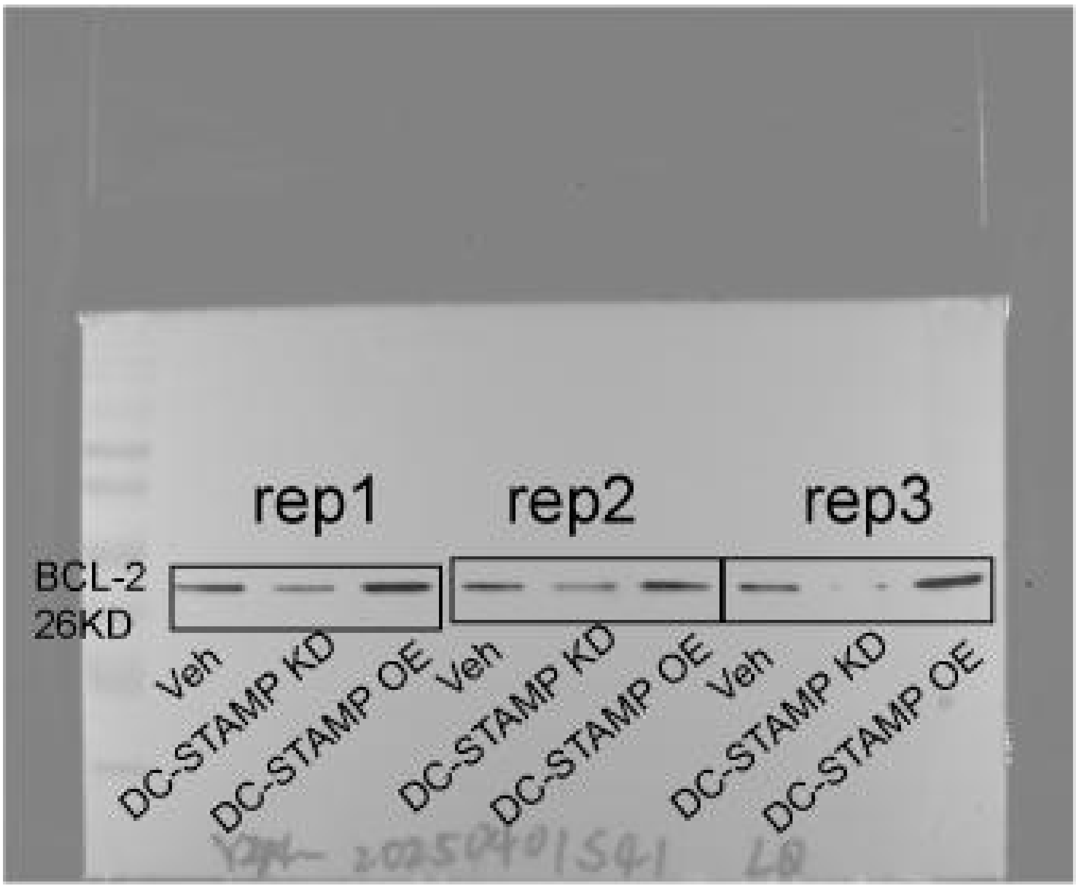
rep1
rep2
rep3
BCL-2
26KD
Veh
DC-STAMP KD
DC-STAMP OE

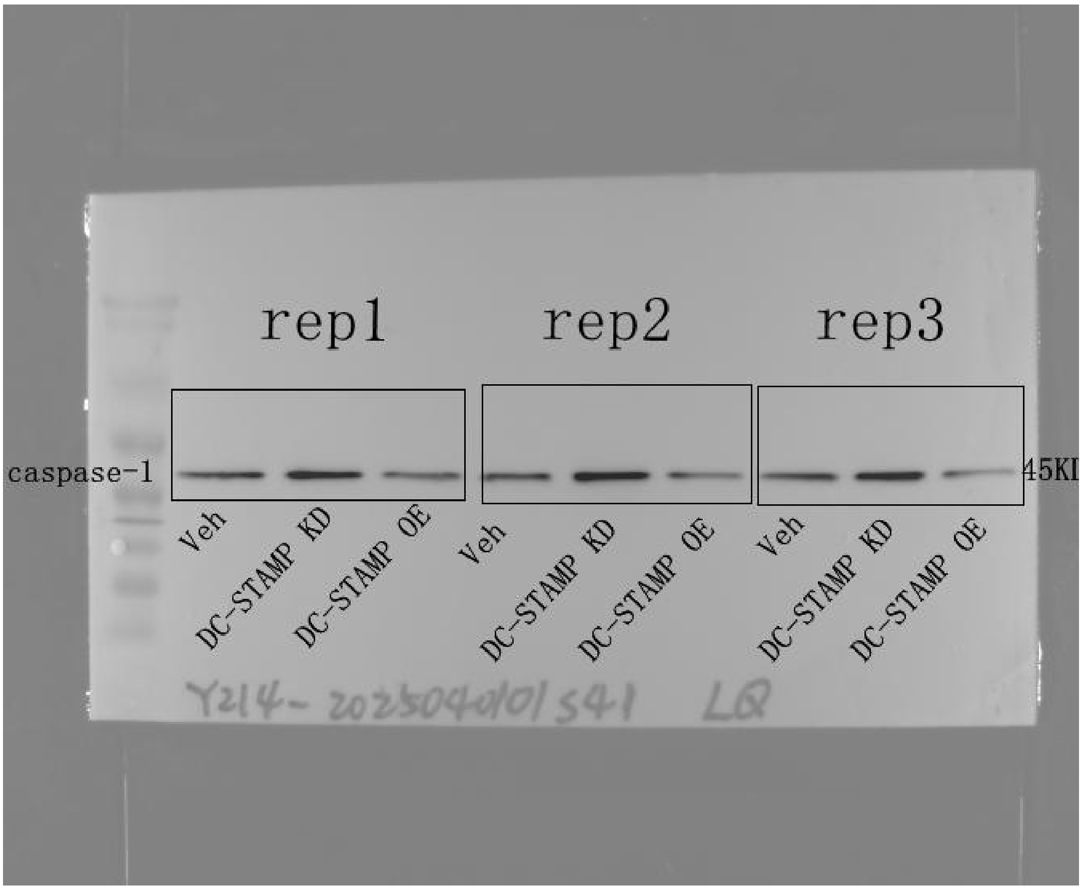
rep1
rep2
rep3
caspase-1
45KD
Veh
DC-STAMP KD
DC-STAMP OE
Veh
DC-STAMP KD
DC-STAMP OE
Veh
DC-STAMP KD
DC-STAMP OE

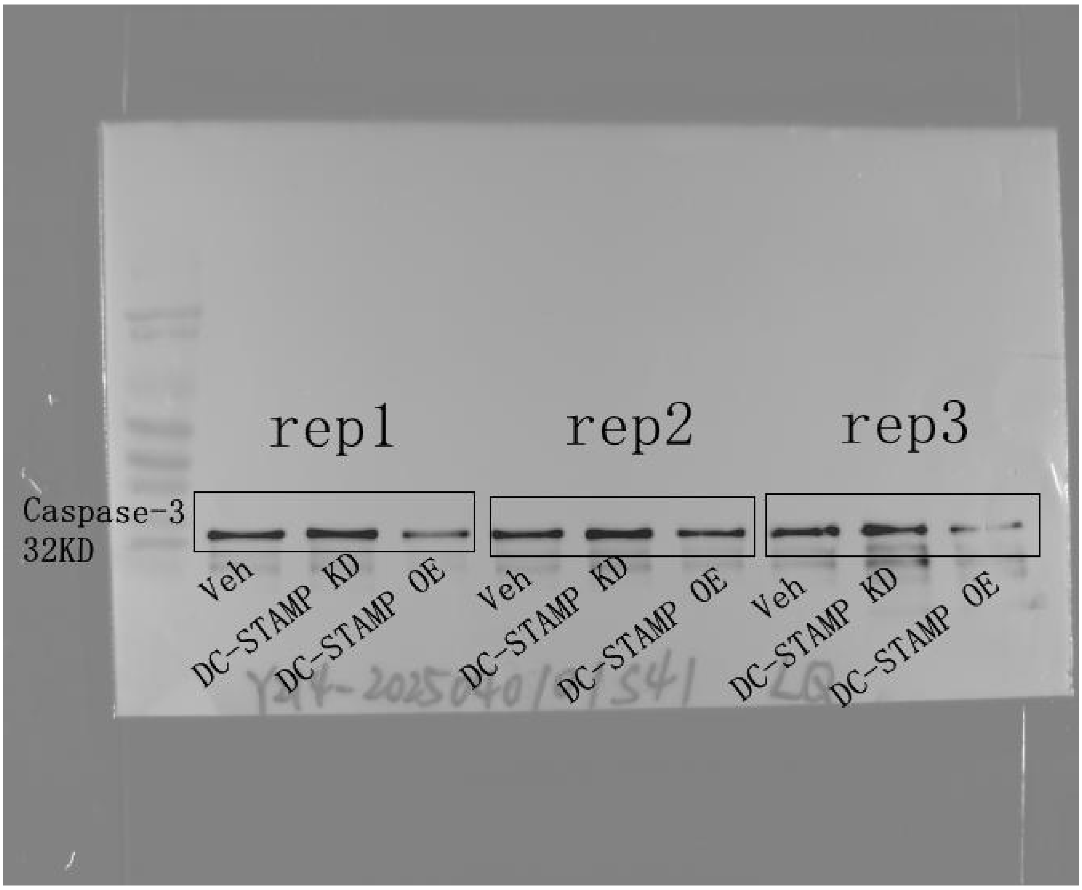
rep1
rep2
rep3
Caspase-3
32KD
Veh
DC-STAMP KD
DC-STAMP OE
Veh
DC-STAMP KD
DC-STAMP OE
Veh
DC-STAMP KD
DC-STAMP OE

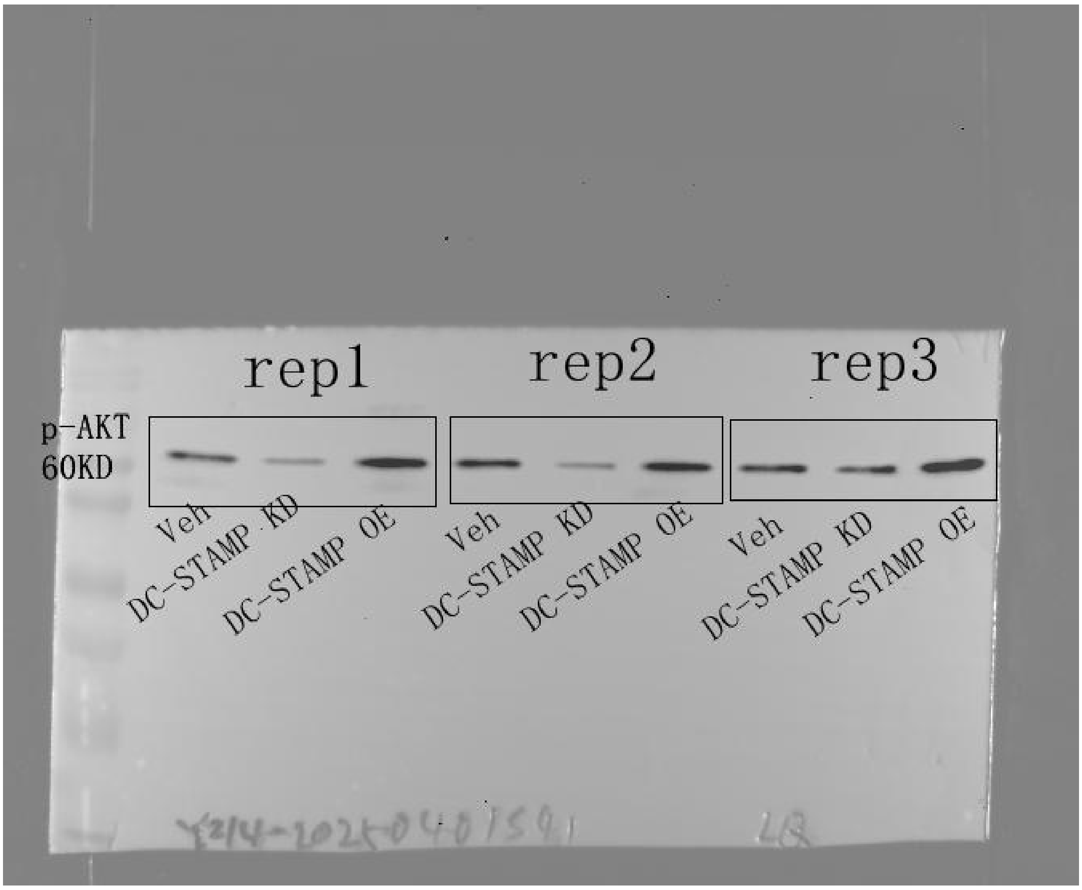
rep1
rep2
rep3
p-AKT
60KD
Veh
DC-STAMP KD
DC-STAMP OE
Veh
DC-STAMP KD
DC-STAMP OE
Veh
DC-STAMP KD
DC-STAMP OE

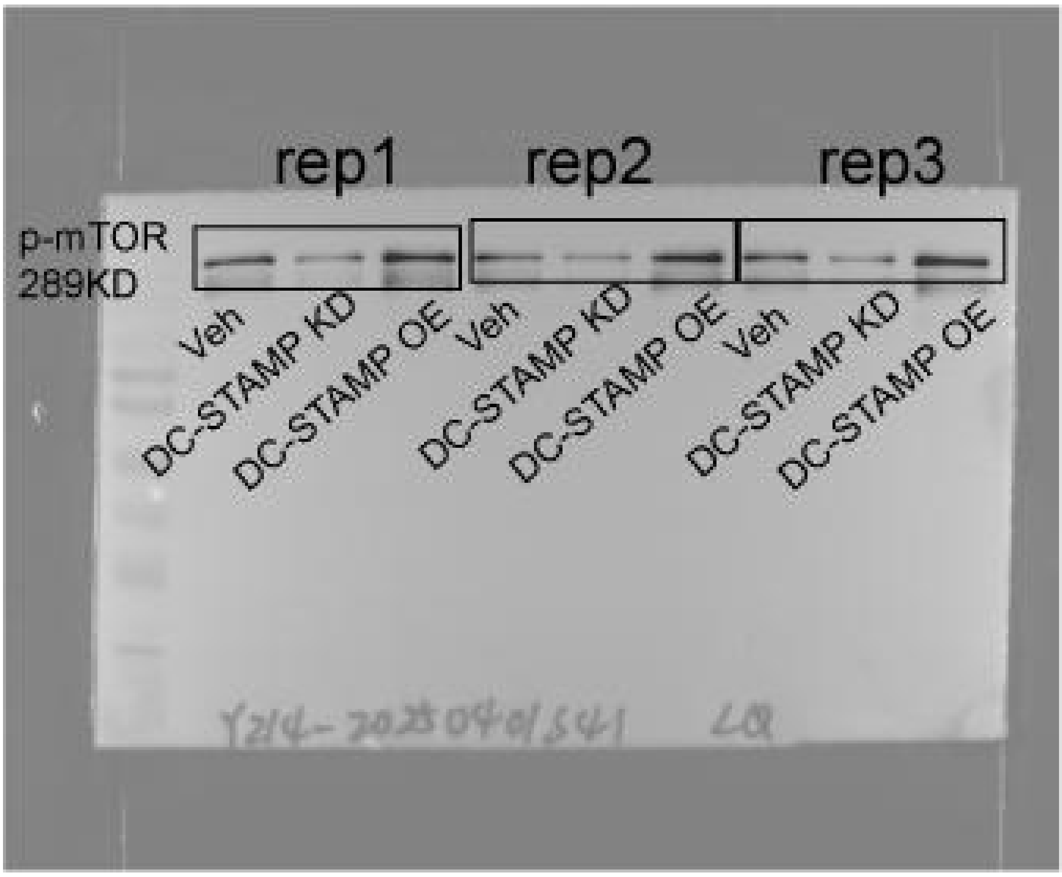
rep1
rep2
rep3
p-mTOR
289KD
Veh
DC-STAMP KD
DC-STAMP OE
Veh
DC-STAMP KD
DC-STAMP OE
Veh
DC-STAMP KD
DC-STAMP OE

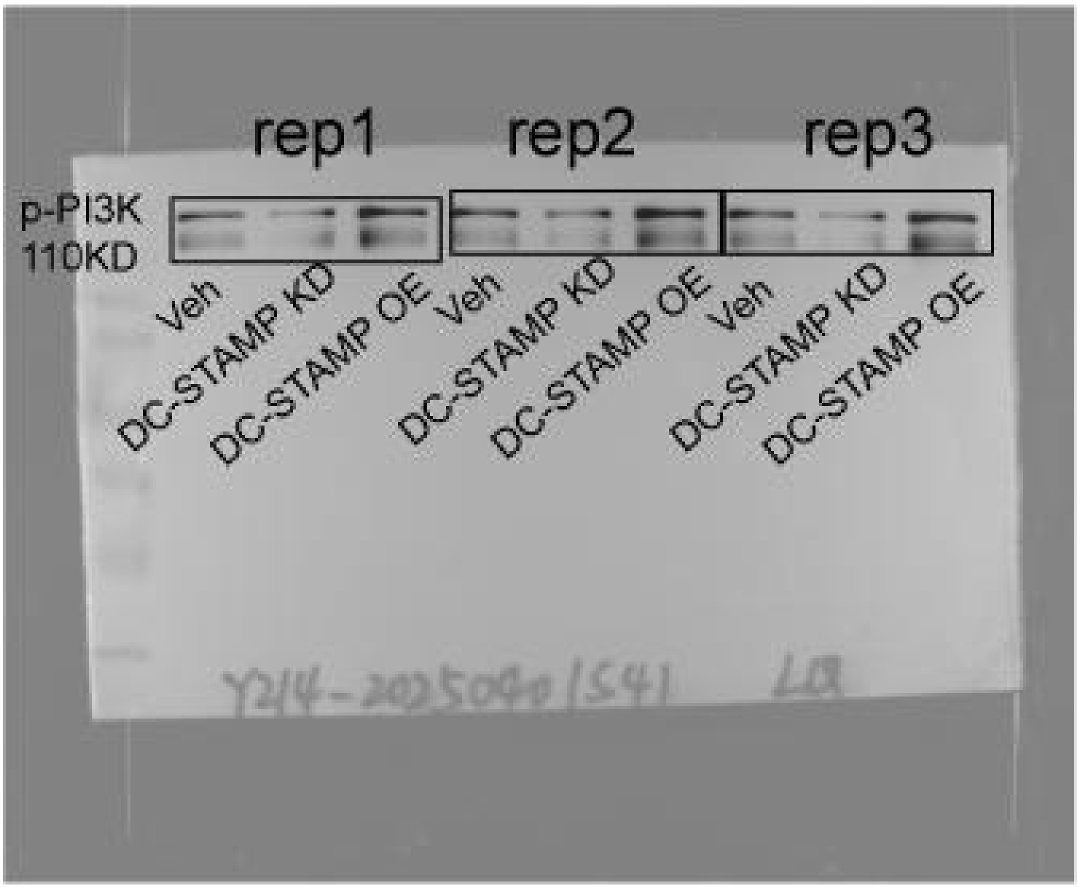
rep1
rep2
rep3
p-PI3K
110KD
Veh
DC-STAMP KD
DC-STAMP OE
Veh
DC-STAMP KD
DC-STAMP OE
Veh
DC-STAMP KD
DC-STAMP OE

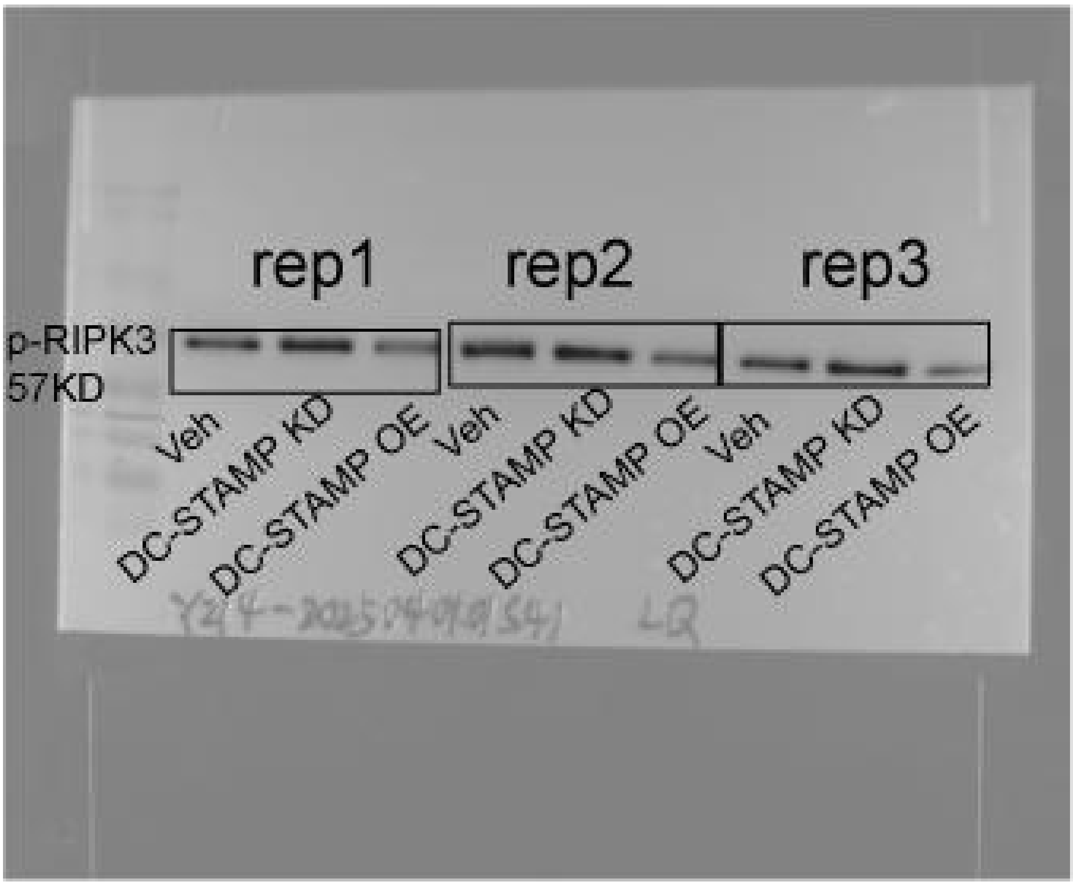
rep1
rep2
rep3
p-RIPK3
57KD
Veh
DC-STAMP KD
DC-STAMP OE
Veh
DC-STAMP KD
DC-STAMP OE
Veh
DC-STAMP KD
DC-STAMP OE

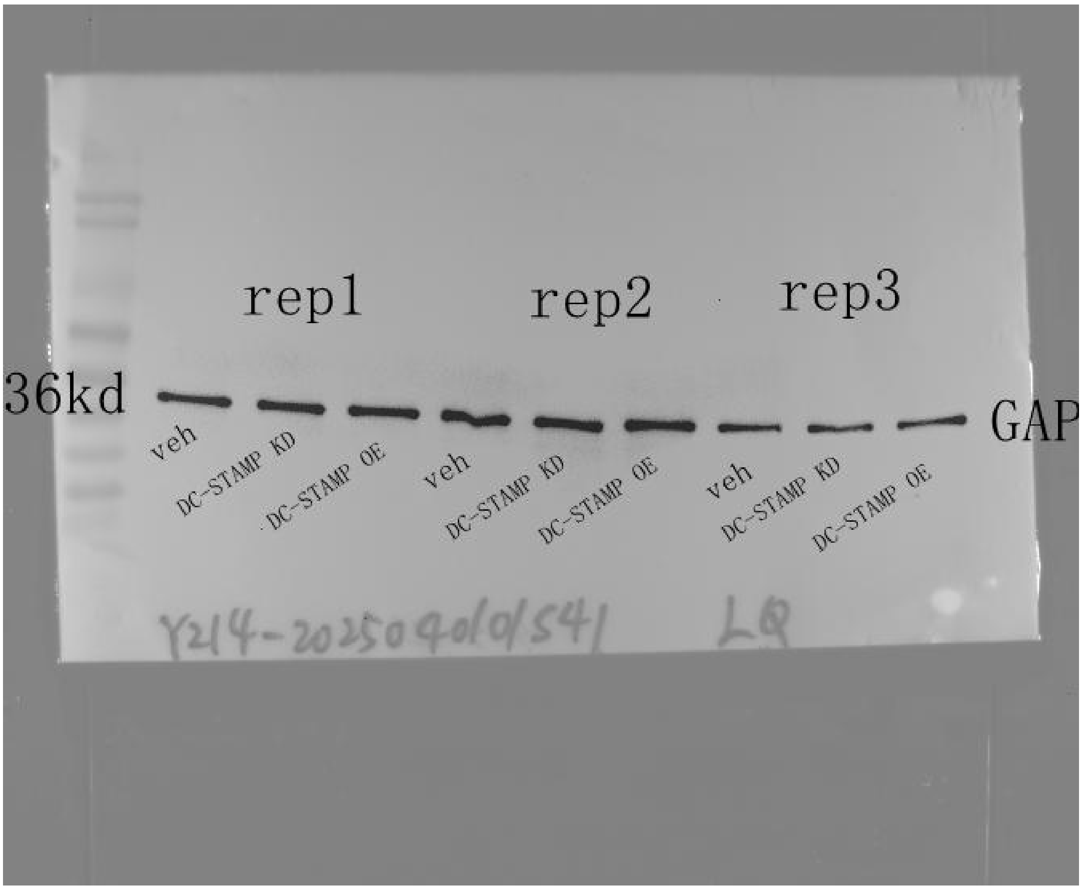
rep1
rep2
rep3
36kd
GAP
veh
DC-STAMP KD
DC-STAMP OE
veh
DC-STAMP KD
DC-STAMP OE
veh
DC-STAMP KD
DC-STAMP OE

Fig5c

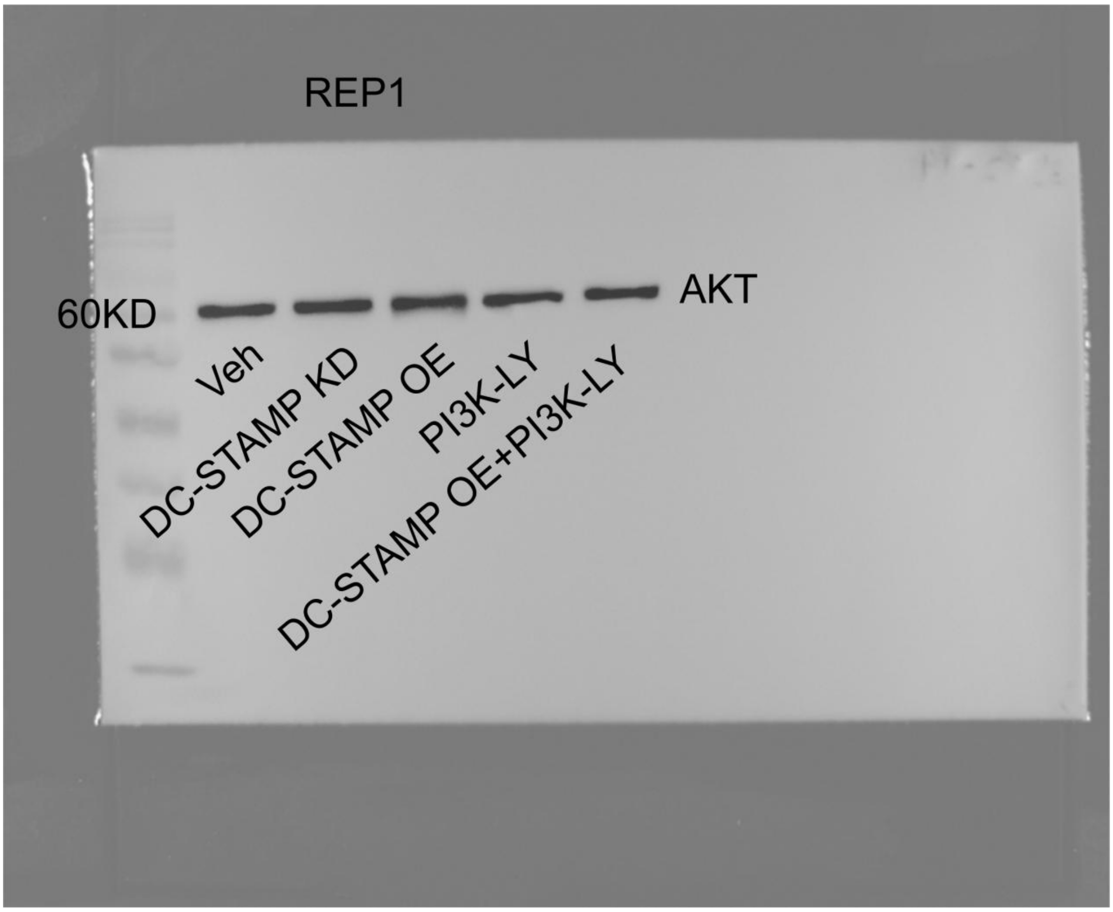
REP1
60KD
AKT
Veh
DC-STAMP KD
DC-STAMP OE
PI3K-LY
DC-STAMP OE+PI3K-LY

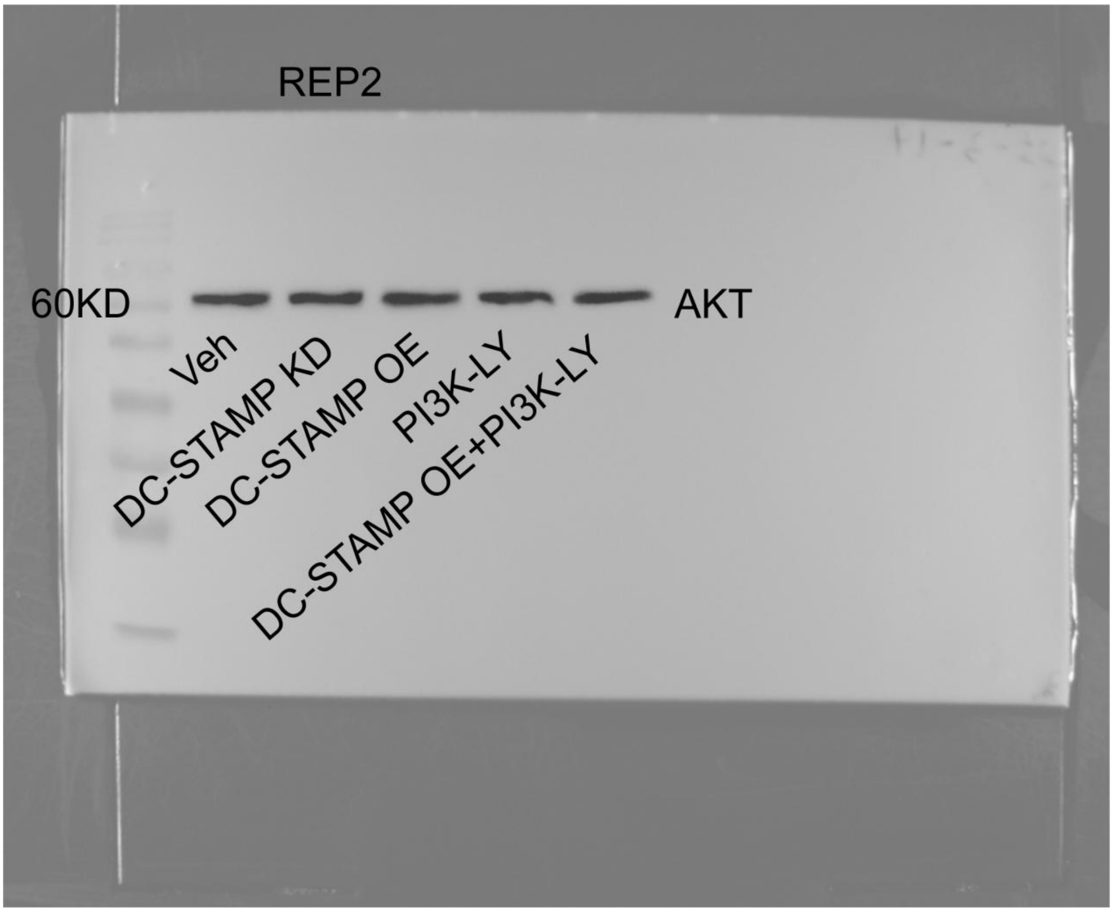
REP2
60KD
AKT
Veh
DC-STAMP KD
DC-STAMP OE
PI3K-LY
DC-STAMP OE+PI3K-LY

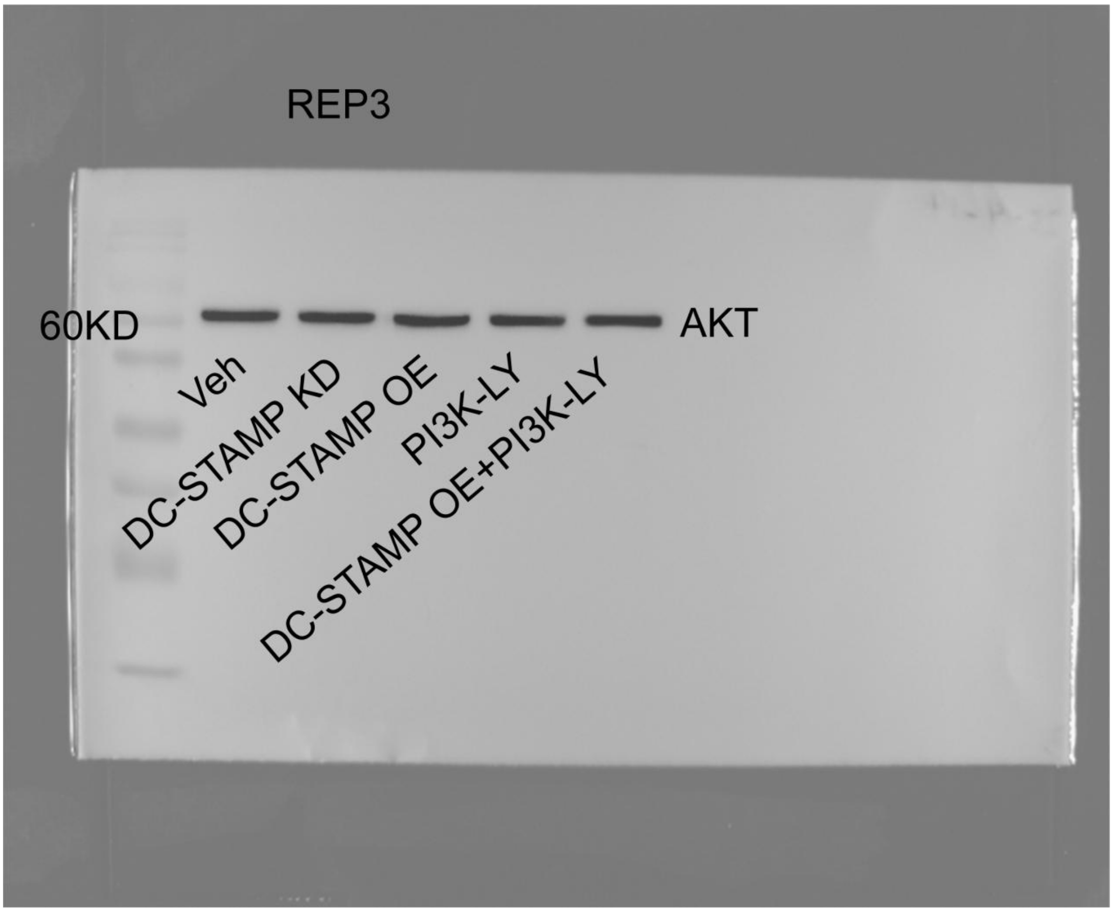
REP3
60KD
AKT
Veh
DC-STAMP KD
DC-STAMP OE
PI3K-LY
DC-STAMP OE+PI3K-LY

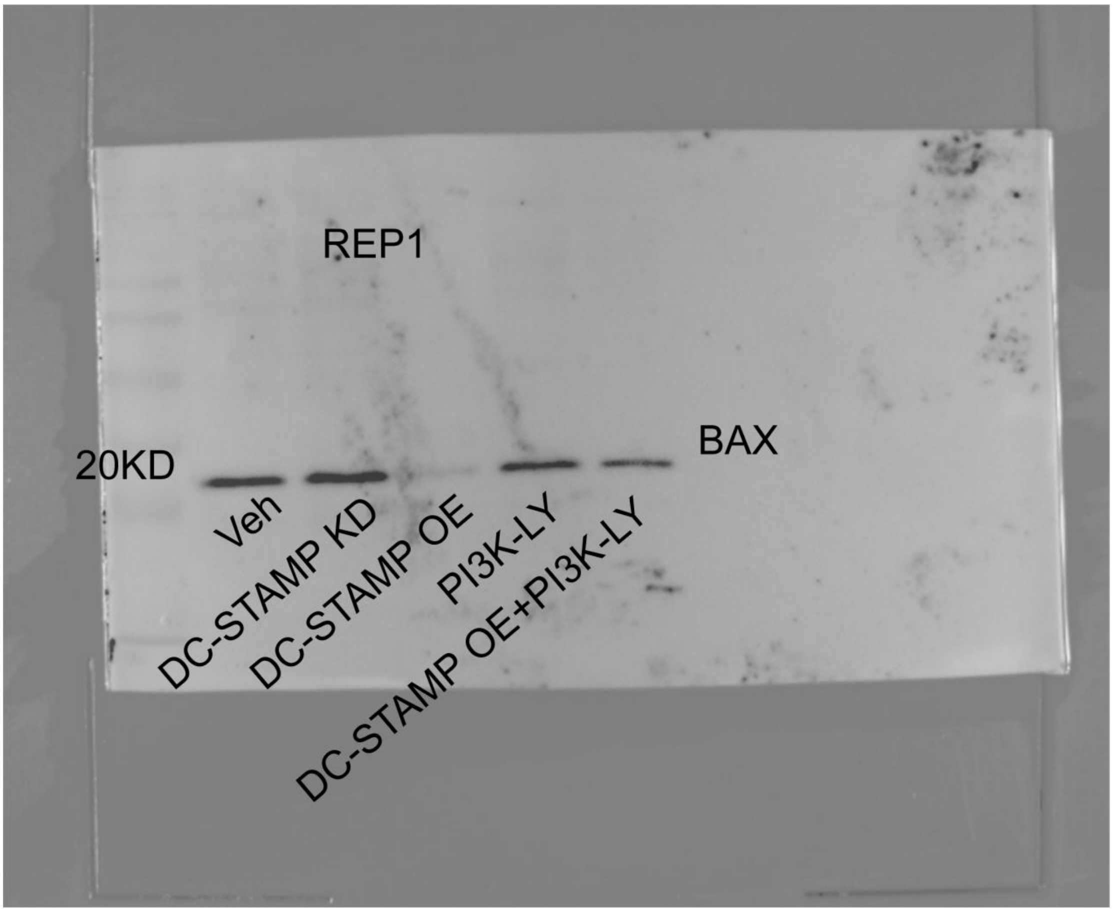
REP1
20KD
BAX
Veh
DC-STAMP KD
DC-STAMP OE
PI3K-LY
DC-STAMP OE+PI3K-LY

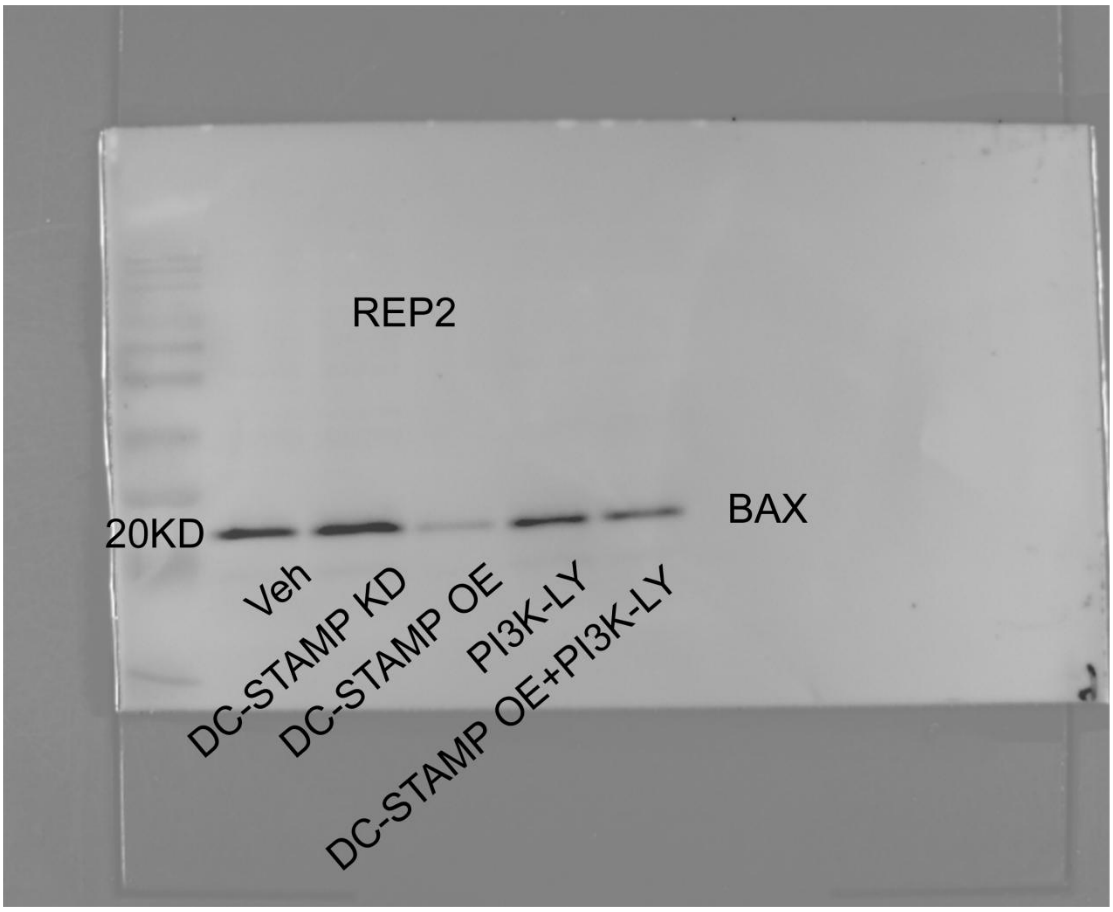
REP2
20KD
BAX
Veh
DC-STAMP KD
DC-STAMP OE
PI3K-LY
DC-STAMP OE+PI3K-LY

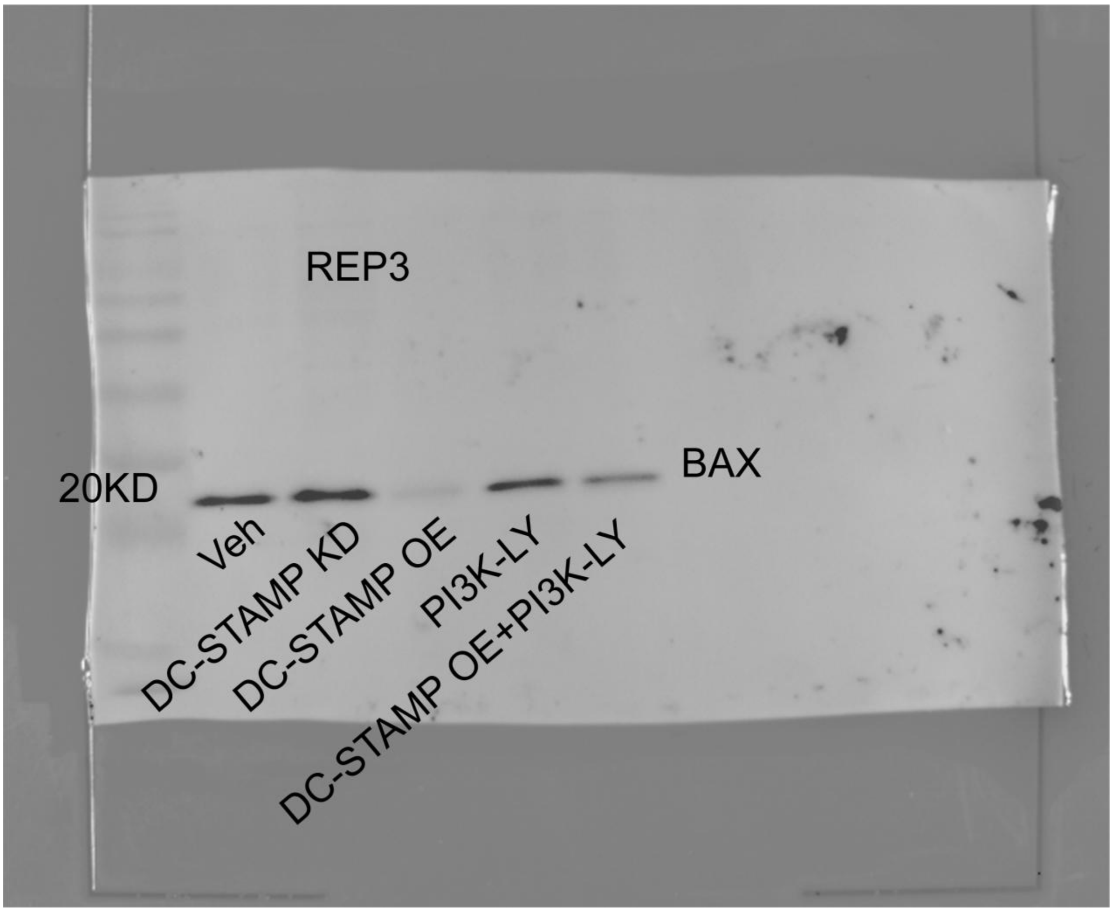
REP3
20KD
BAX
Veh
DC-STAMP KD
DC-STAMP OE
PI3K-LY
DC-STAMP OE+PI3K-LY

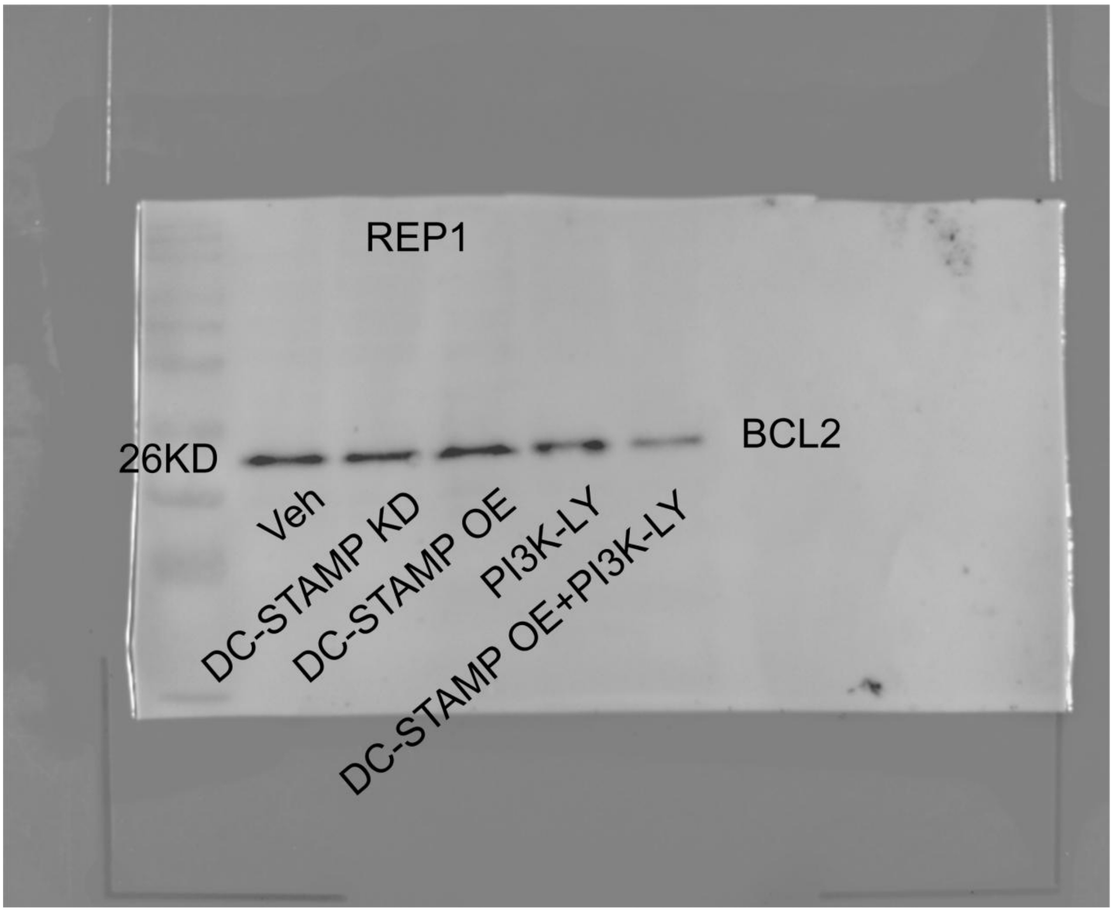
REP1
BCL2
26KD
Veh
DC-STAMP KD
DC-STAMP OE
PI3K-LY
DC-STAMP OE+PI3K-LY

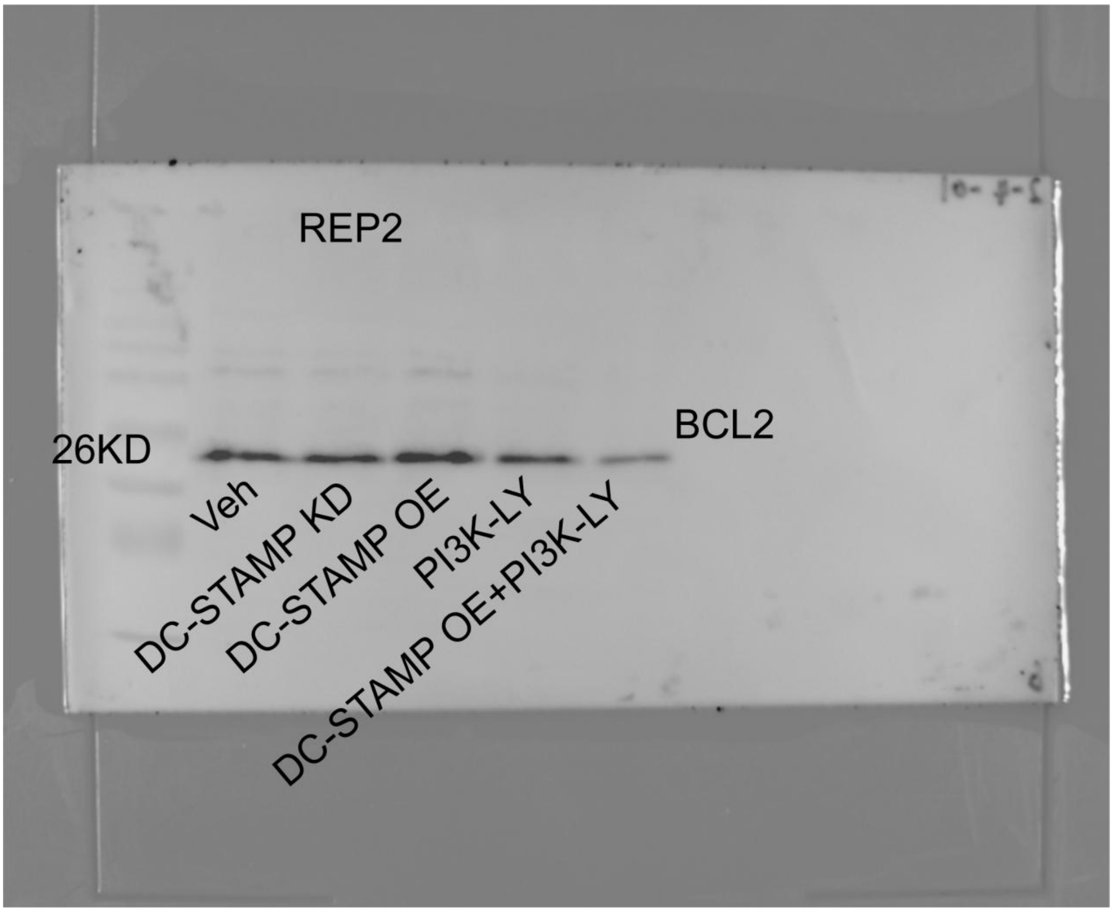
REP2
BCL2
26KD
Veh
DC-STAMP KD
DC-STAMP OE
PI3K-LY
DC-STAMP OE+PI3K-LY

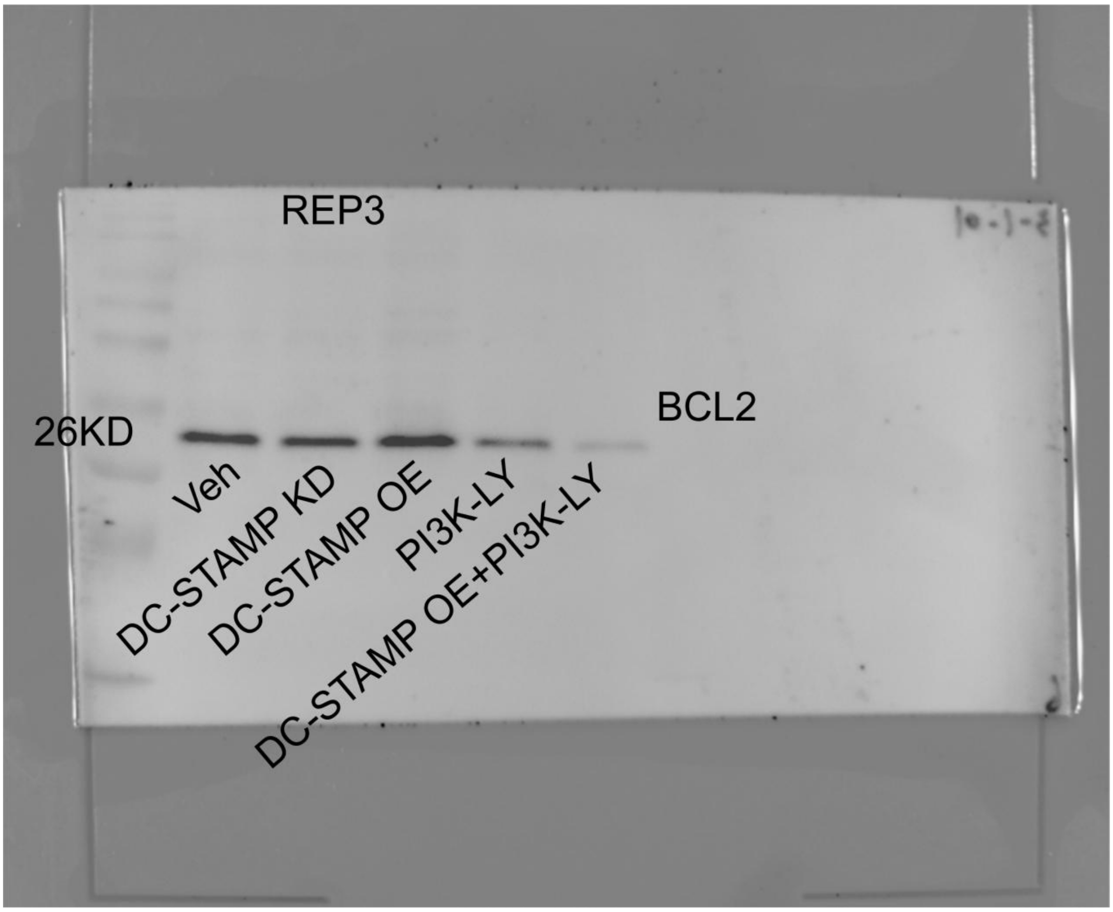
REP3
BCL2
26KD
Veh
DC-STAMP KD
DC-STAMP OE
PI3K-LY
DC-STAMP OE+PI3K-LY

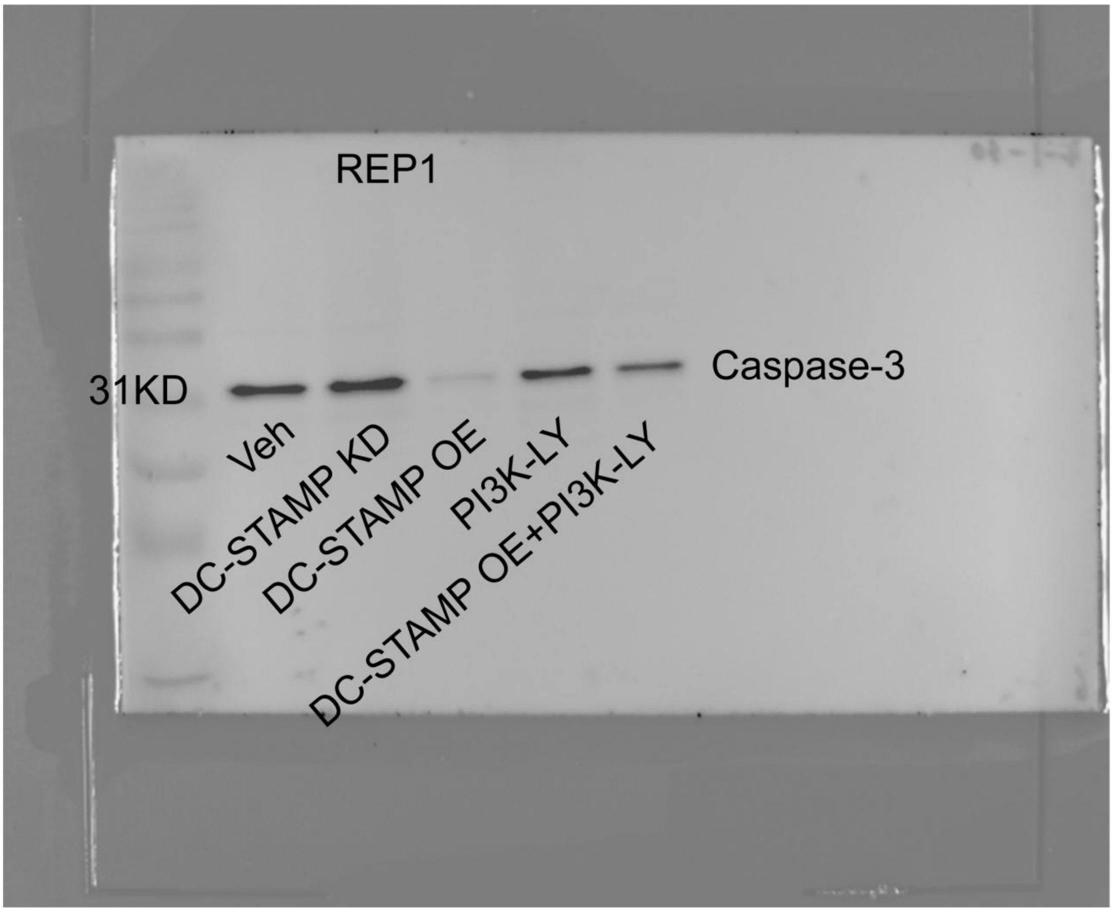
REP1
Caspase-3
31KD
Veh
DC-STAMP KD
DC-STAMP OE
PI3K-LY
DC-STAMP OE+PI3K-LY

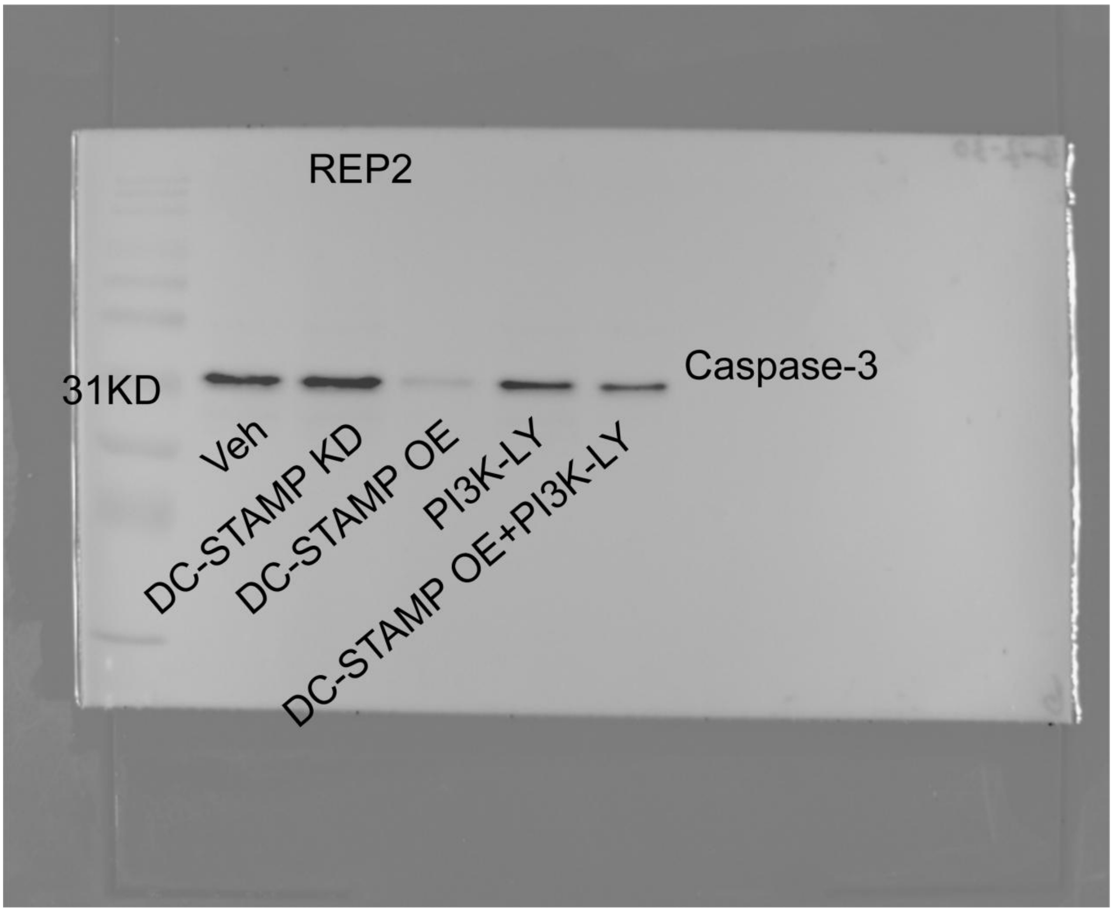
REP2
31KD
Caspase-3
Veh
DC-STAMP KD
DC-STAMP OE
PI3K-LY
DC-STAMP OE+PI3K-LY

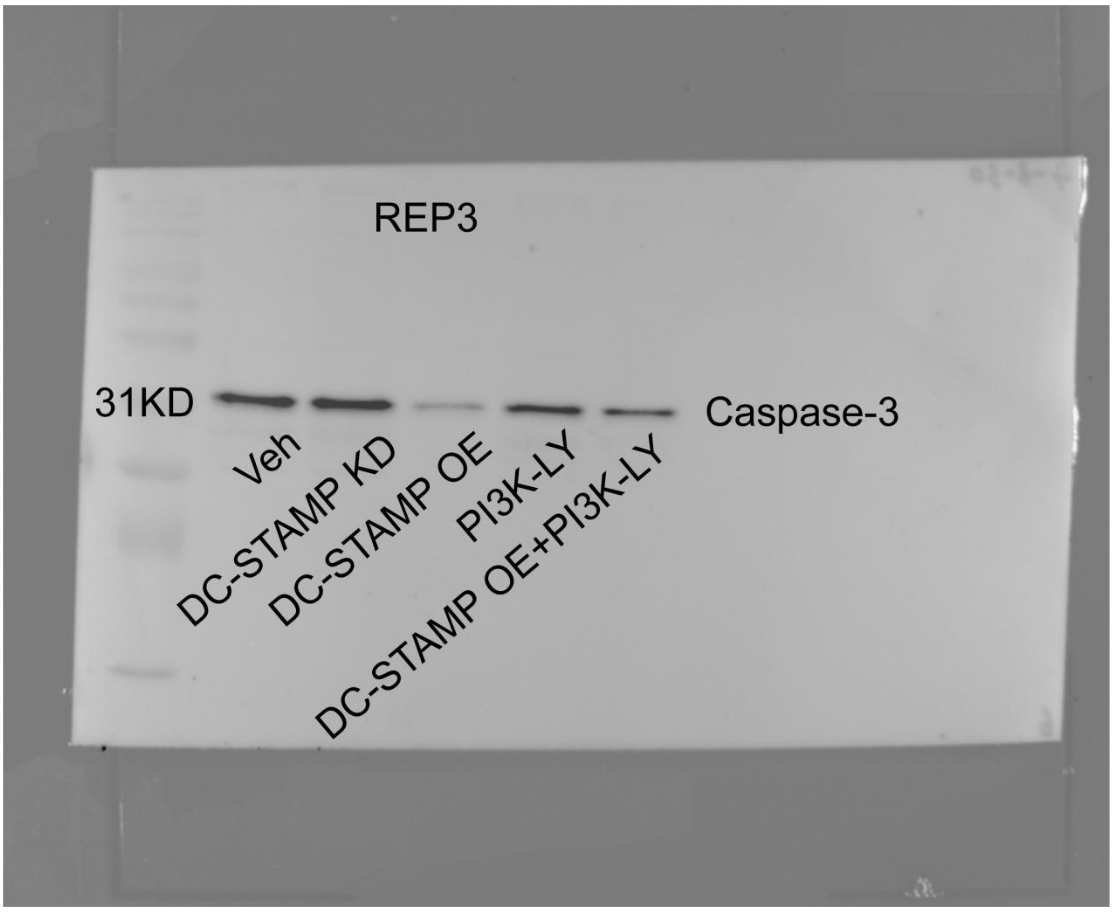
REP3
31KD
Caspase-3
Veh
DC-STAMP KD
DC-STAMP OE
PI3K-LY
DC-STAMP OE+PI3K-LY

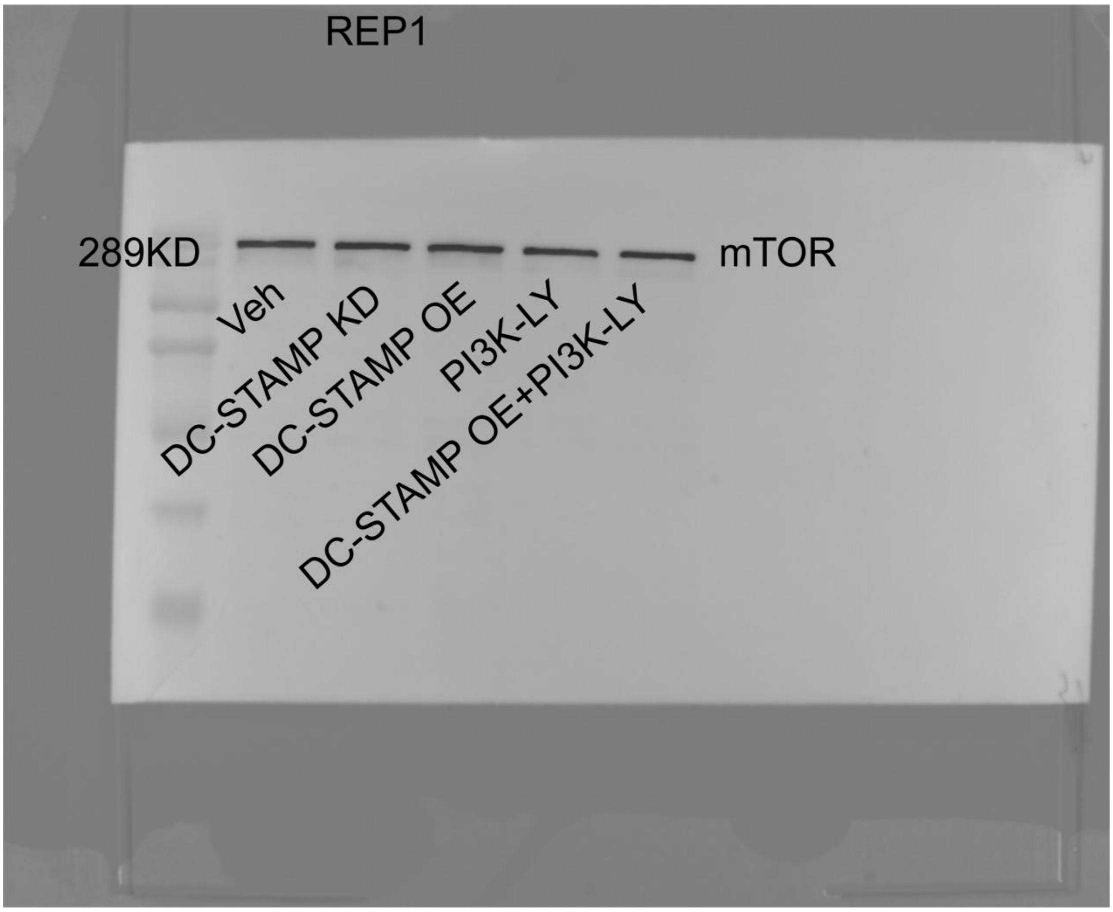
REP1
289KD
mTOR
Veh
DC-STAMP KD
DC-STAMP OE
PI3K-LY
DC-STAMP OE+PI3K-LY

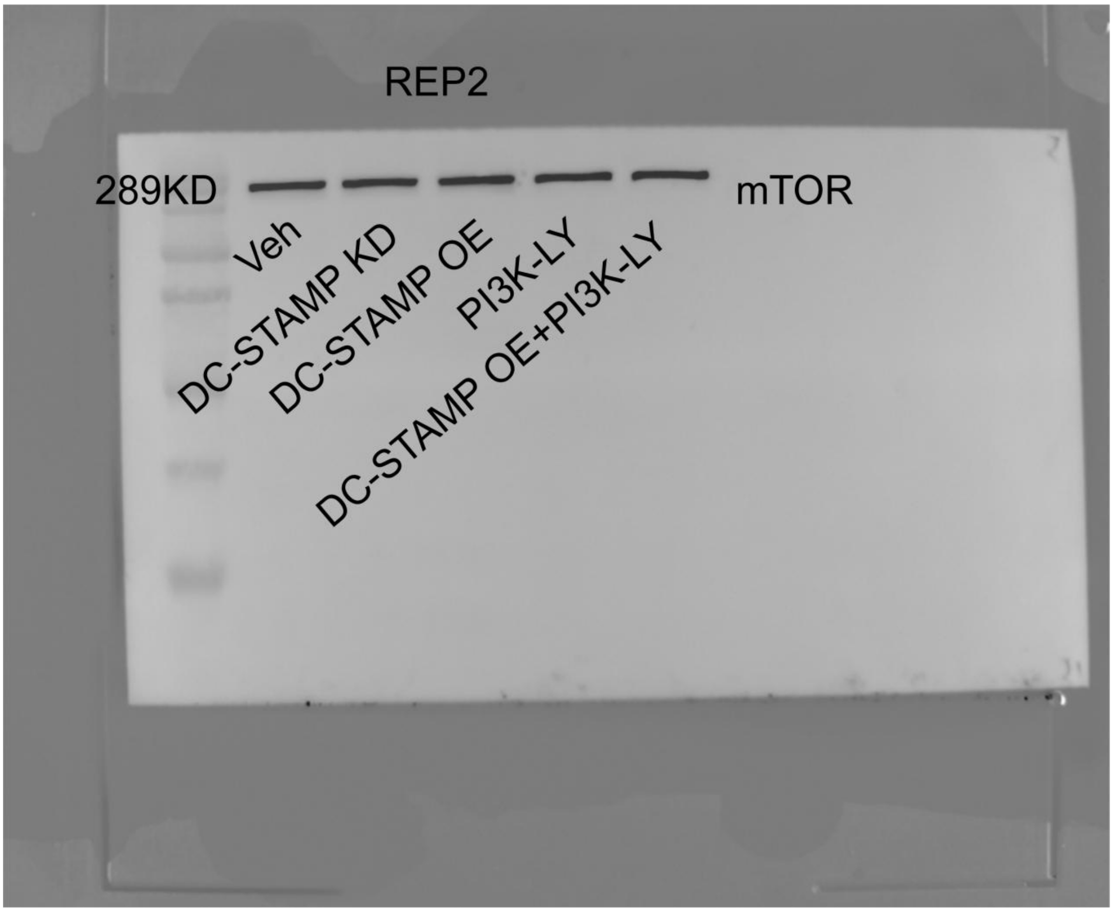
REP2
289KD
mTOR
Veh
DC-STAMP KD
DC-STAMP OE
PI3K-LY
DC-STAMP OE+PI3K-LY

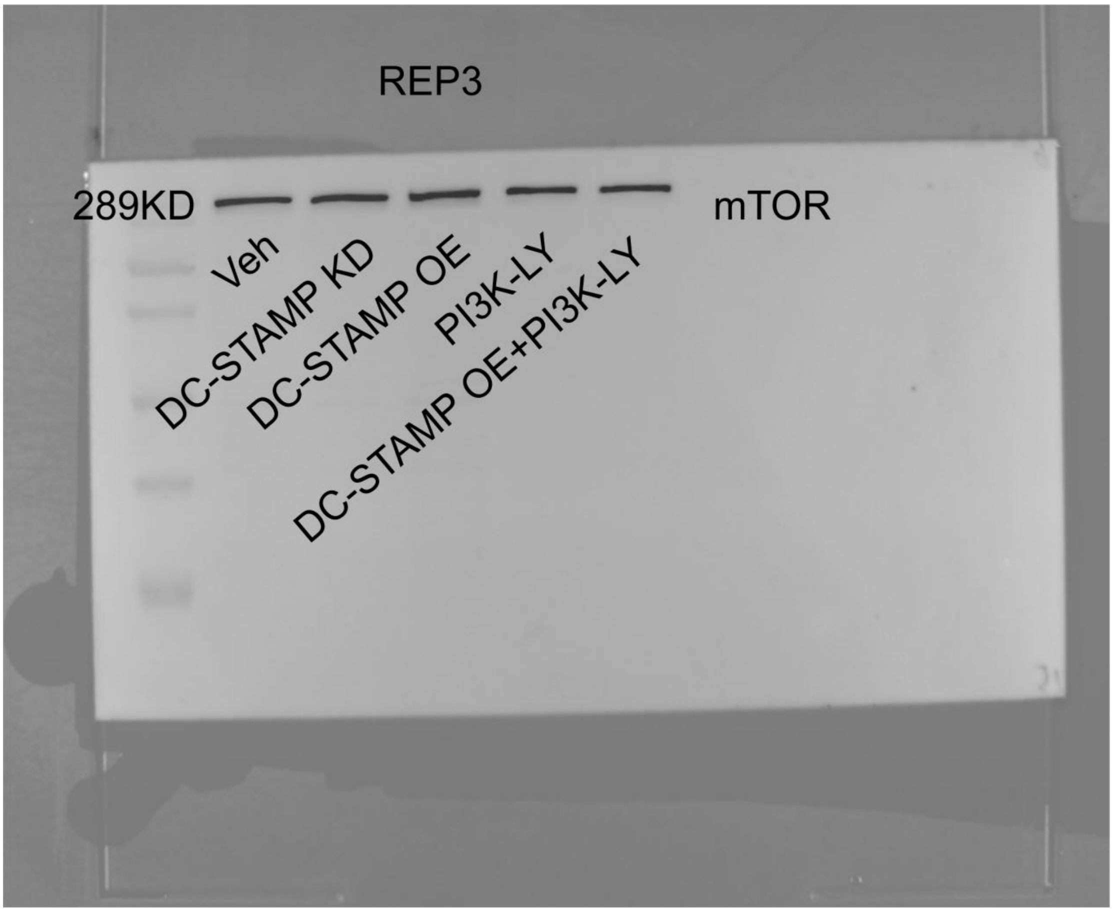
REP3
289KD
mTOR
Veh
DC-STAMP KD
DC-STAMP OE
PI3K-LY
DC-STAMP OE+PI3K-LY

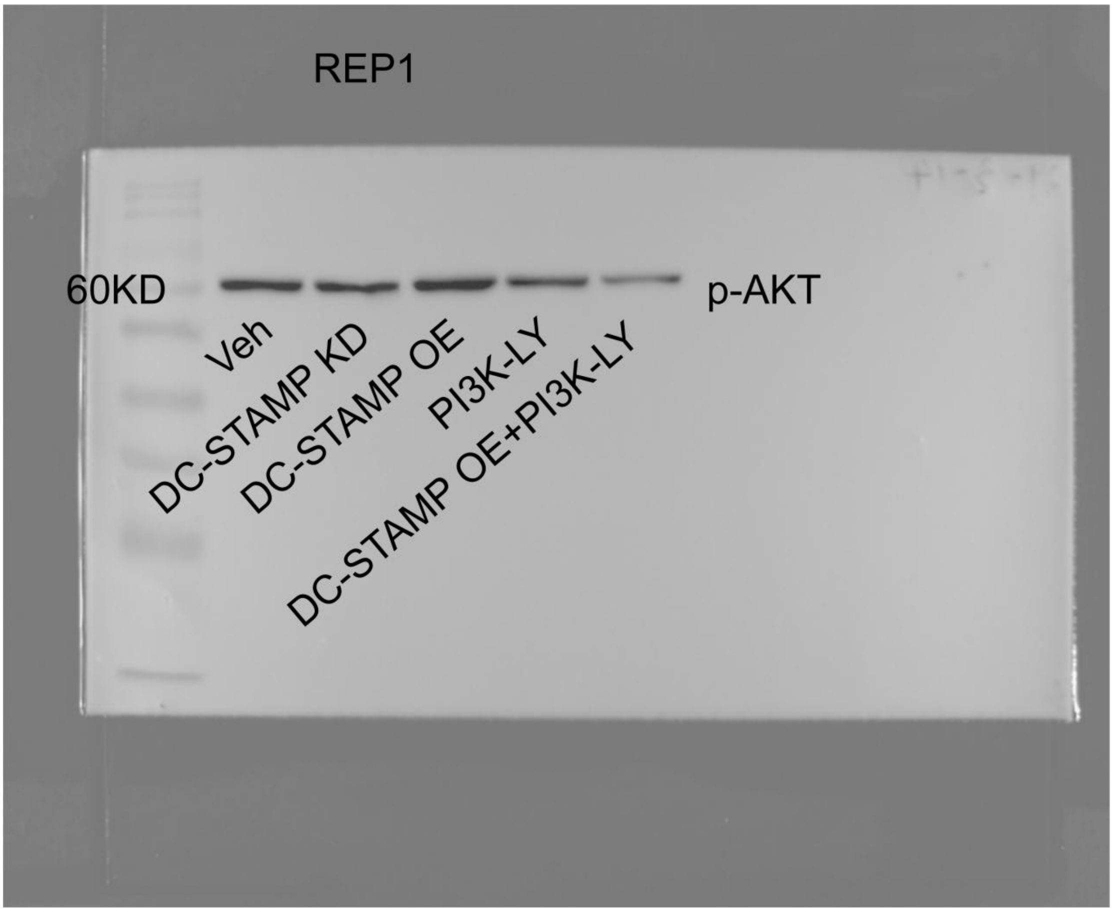
REP1
60KD
p-AKT
Veh
DC-STAMP KD
DC-STAMP OE
PI3K-LY
DC-STAMP OE+PI3K-LY

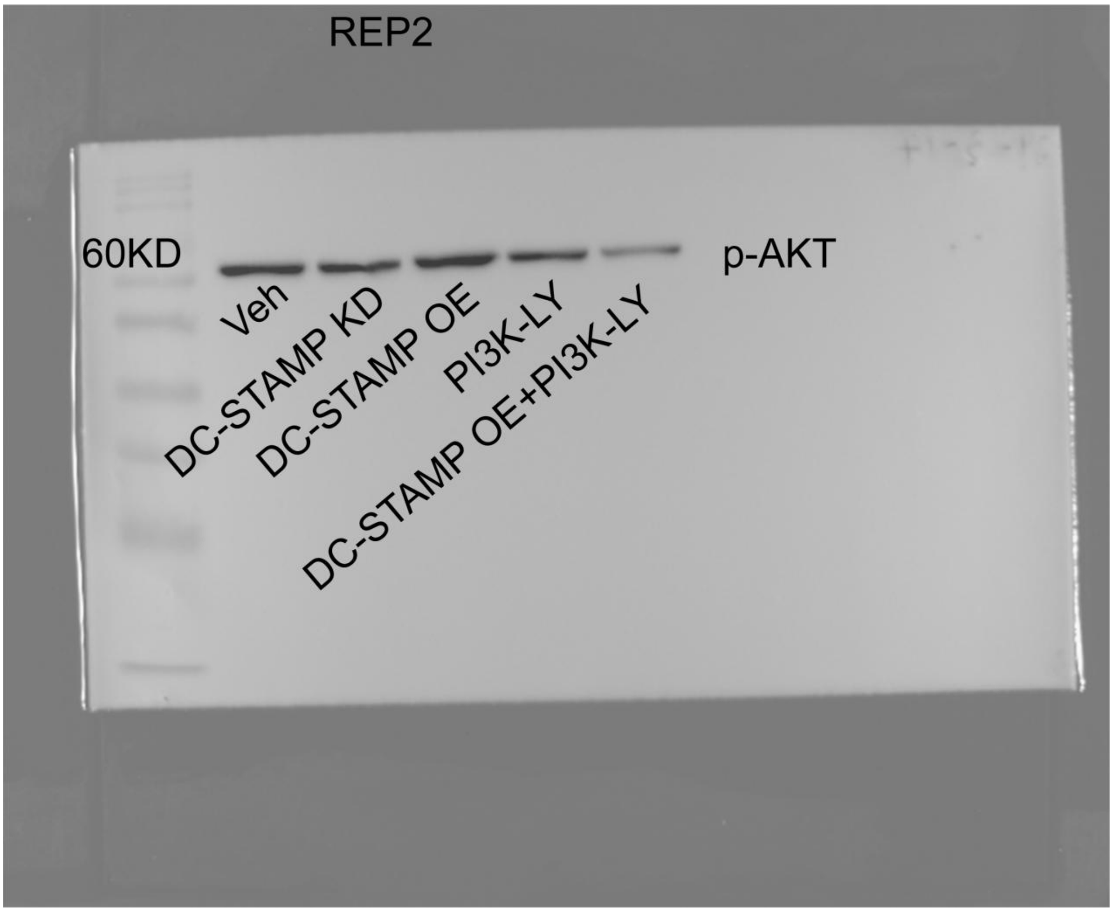
REP2
60KD
p-AKT
Veh
DC-STAMP KD
DC-STAMP OE
PI3K-LY
DC-STAMP OE+PI3K-LY

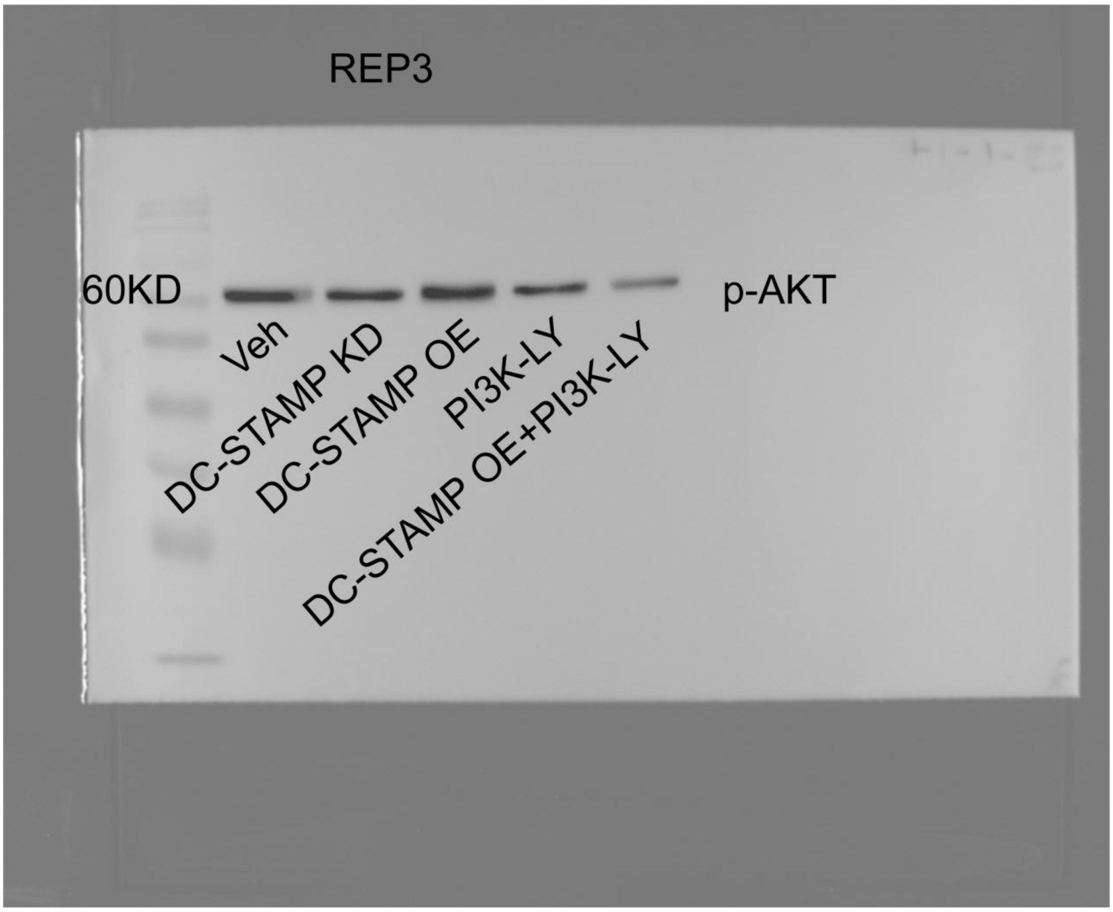
REP3
60KD
p-AKT
Veh
DC-STAMP KD
DC-STAMP OE
PI3K-LY
DC-STAMP OE+PI3K-LY

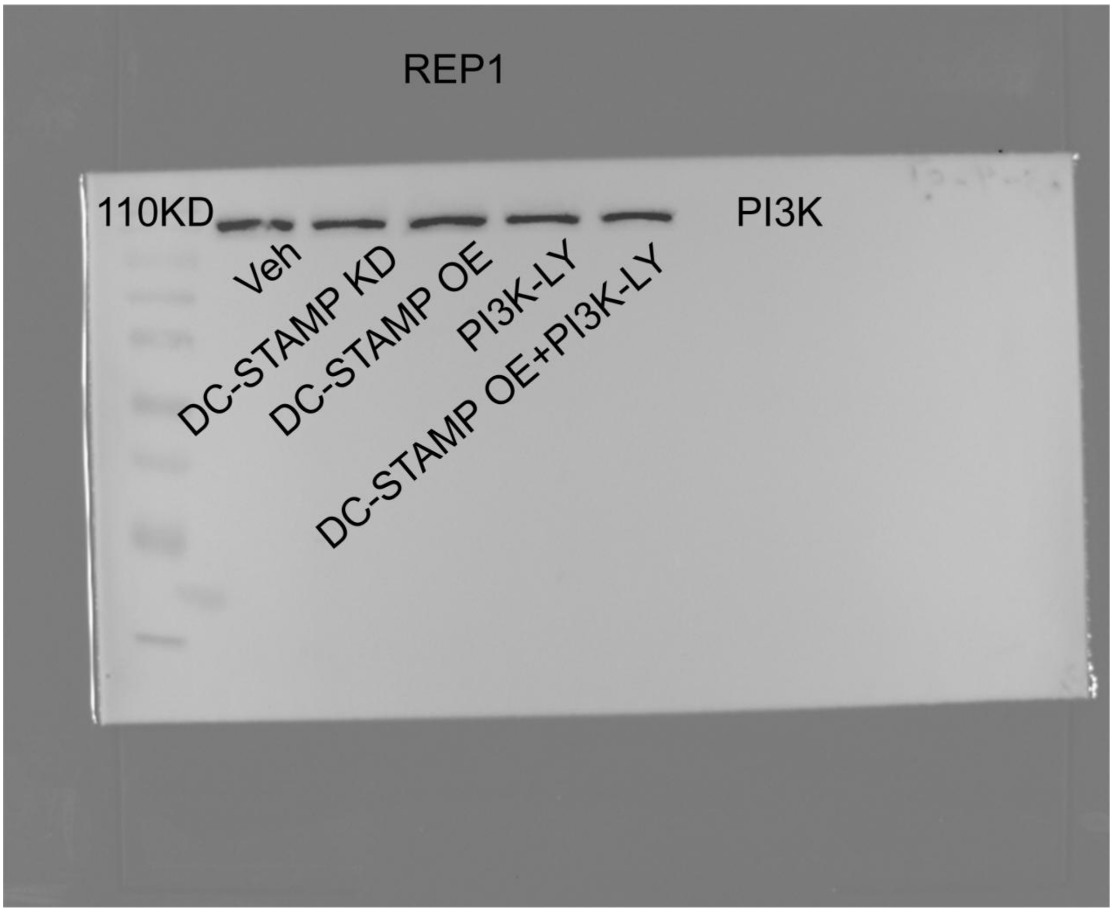
REP1
110KD
PI3K
Veh
DC-STAMP KD
DC-STAMP OE
PI3K-LY
DC-STAMP OE+PI3K-LY

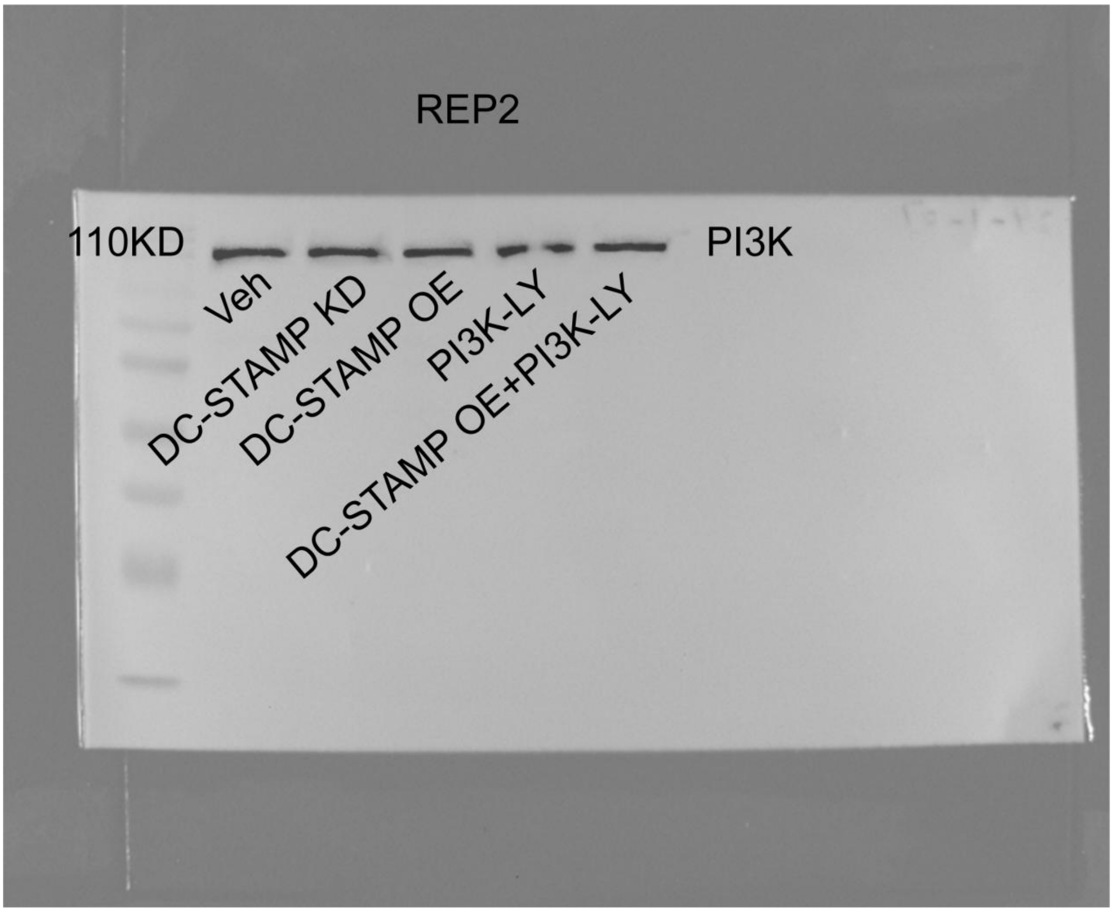
REP2
110KD
PI3K
Veh
DC-STAMP KD
DC-STAMP OE
PI3K-LY
DC-STAMP OE+PI3K-LY

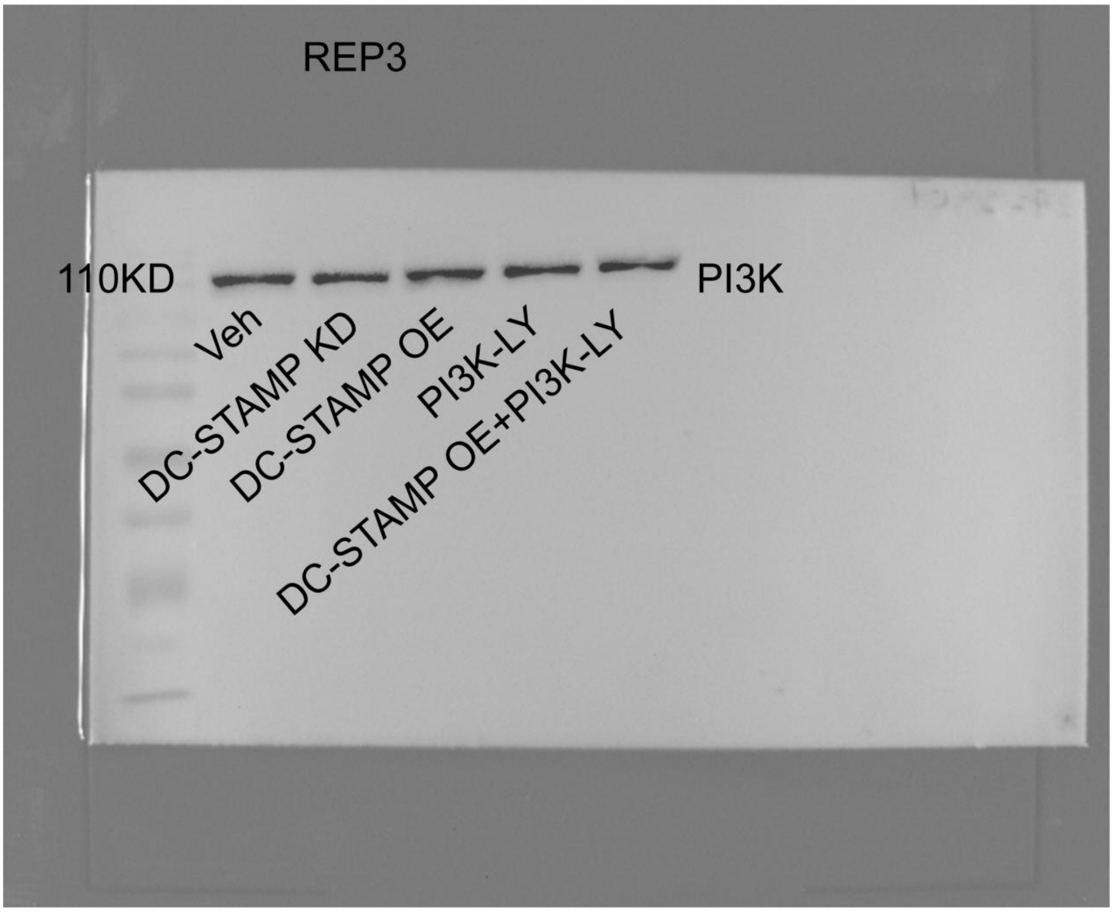
REP3
110KD
PI3K
Veh
DC-STAMP KD
DC-STAMP OE
PI3K-LY
DC-STAMP OE+PI3K-LY

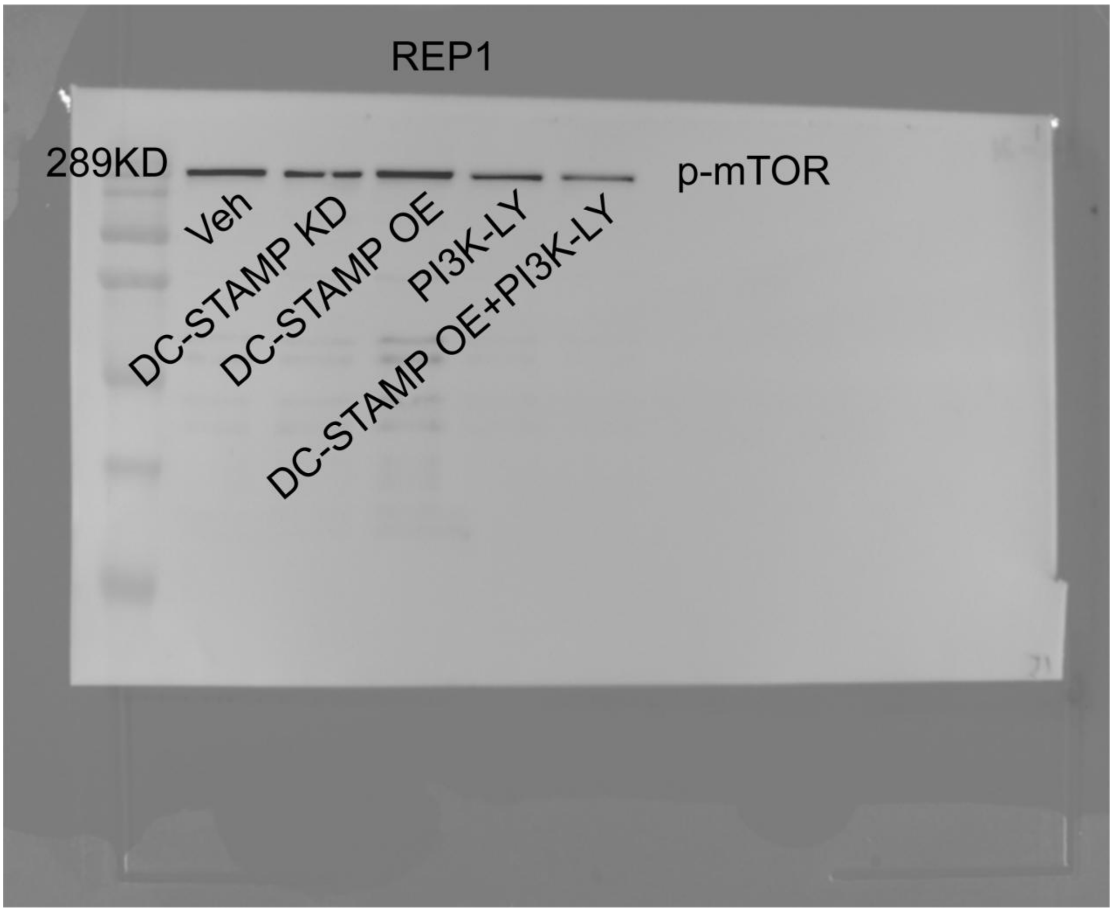
REP1
289KD
p-mTOR
Veh
DC-STAMP KD
DC-STAMP OE
PI3K-LY
DC-STAMP OE+PI3K-LY

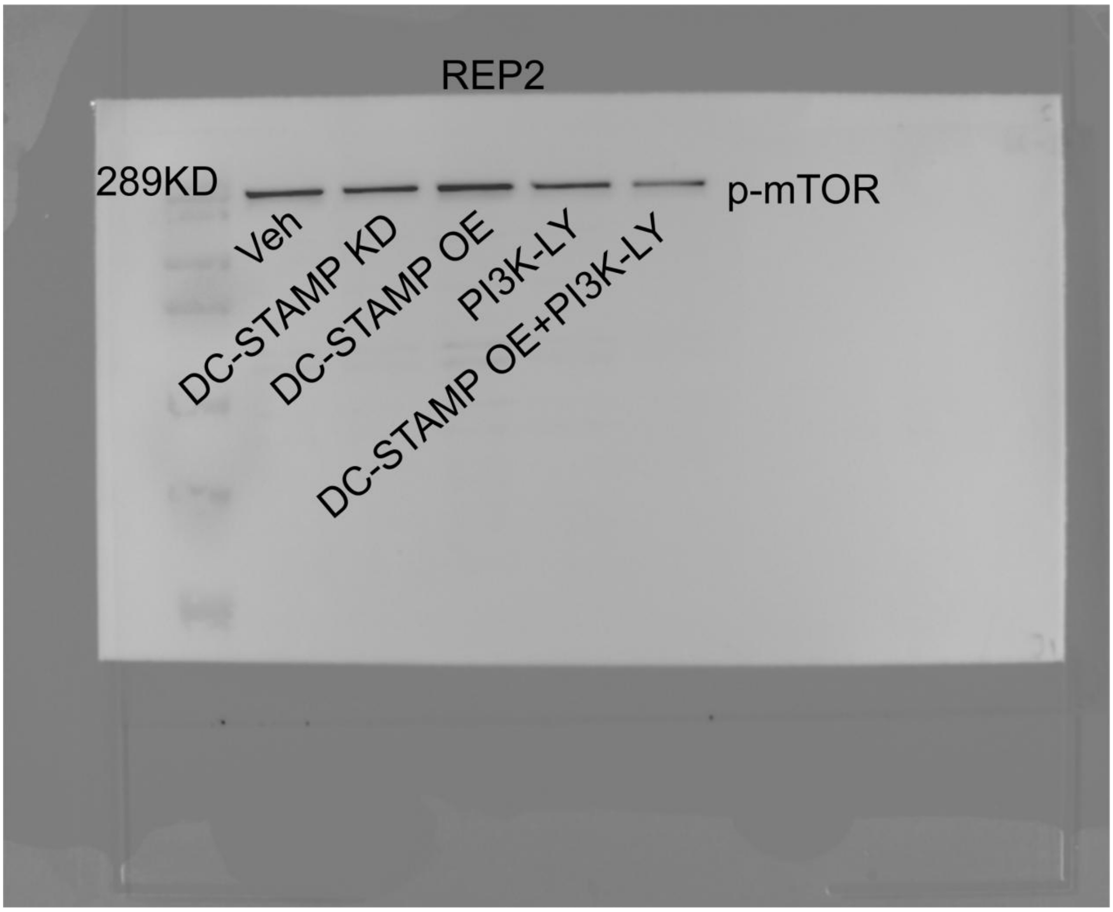
REP2
289KD
p-mTOR
Veh
DC-STAMP KD
DC-STAMP OE
PI3K-LY
DC-STAMP OE+PI3K-LY

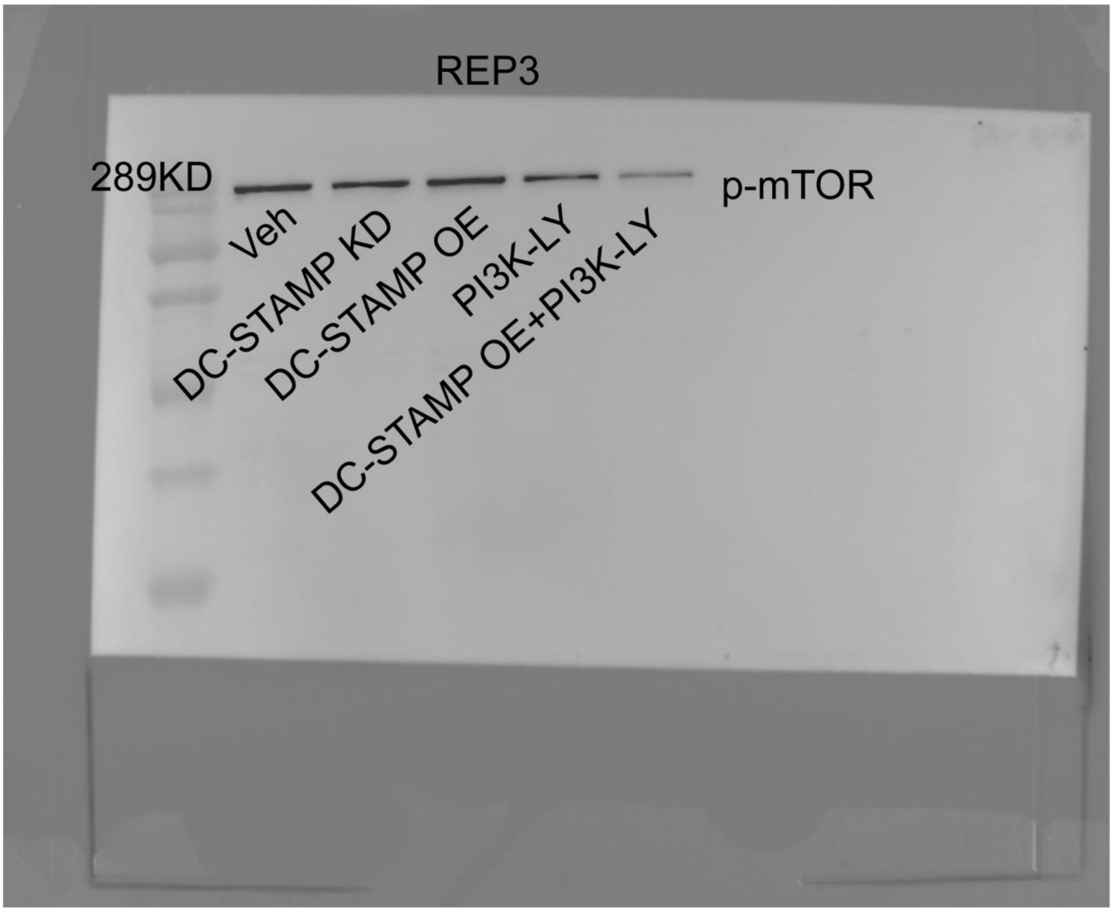
REP3
289KD
p-mTOR
Veh
DC-STAMP KD
DC-STAMP OE
PI3K-LY
DC-STAMP OE+PI3K-LY

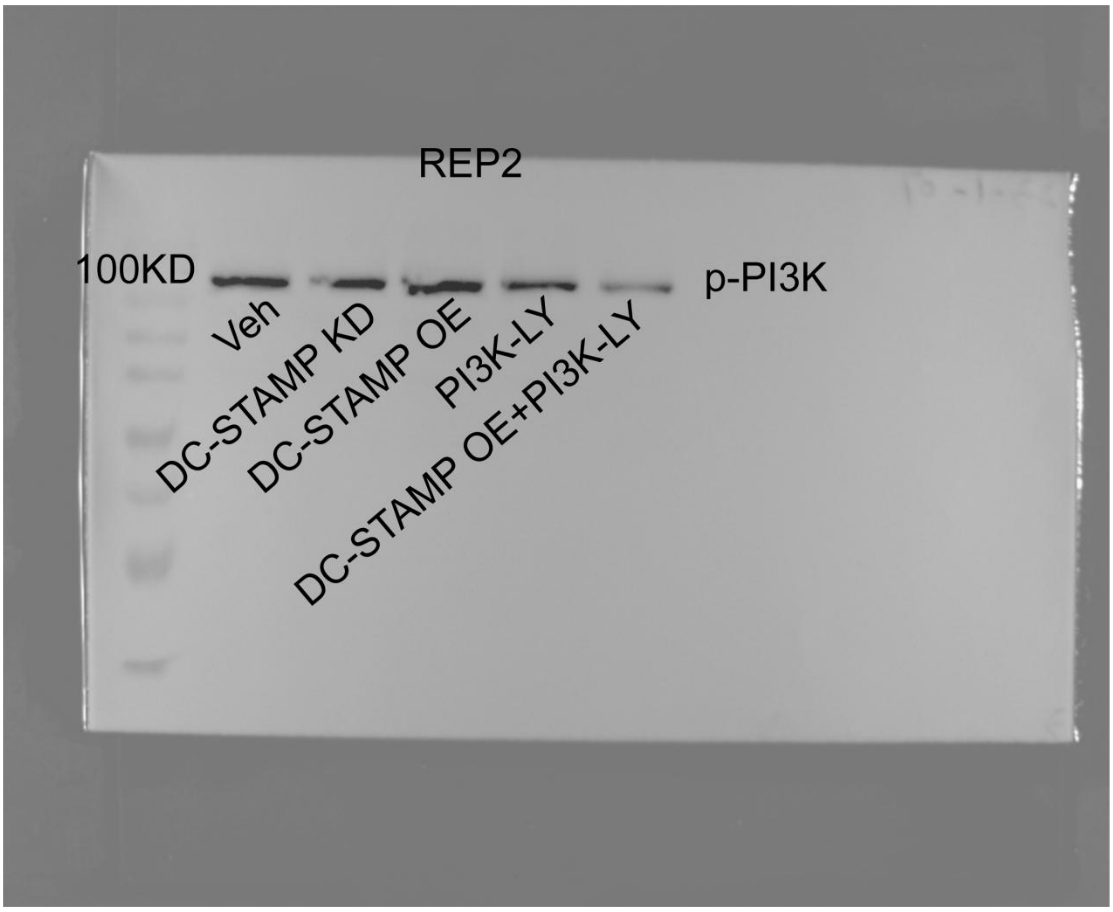
REP2
100KD
p-PI3K
Veh
DC-STAMP KD
DC-STAMP OE
PI3K-LY
DC-STAMP OE+PI3K-LY

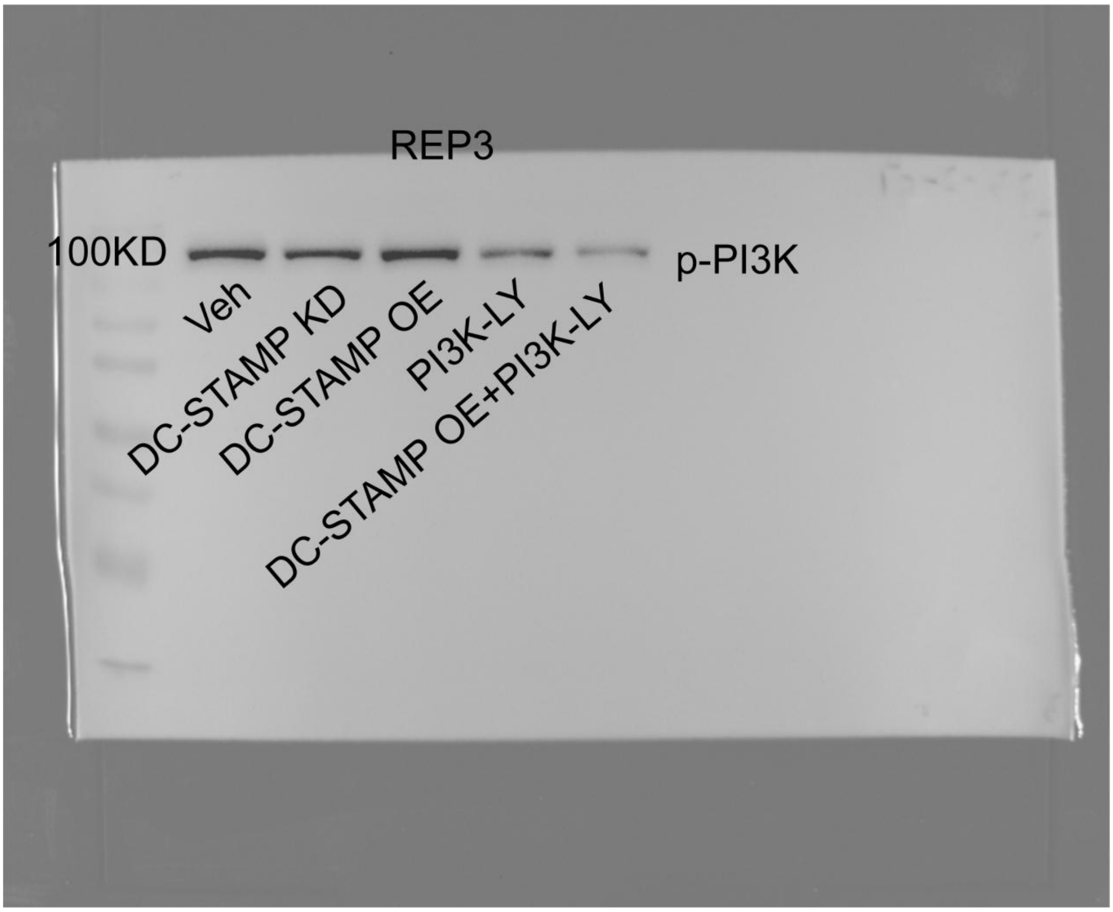
REP3
100KD
p-PI3K
Veh
DC-STAMP KD
DC-STAMP OE
PI3K-LY
DC-STAMP OE+PI3K-LY

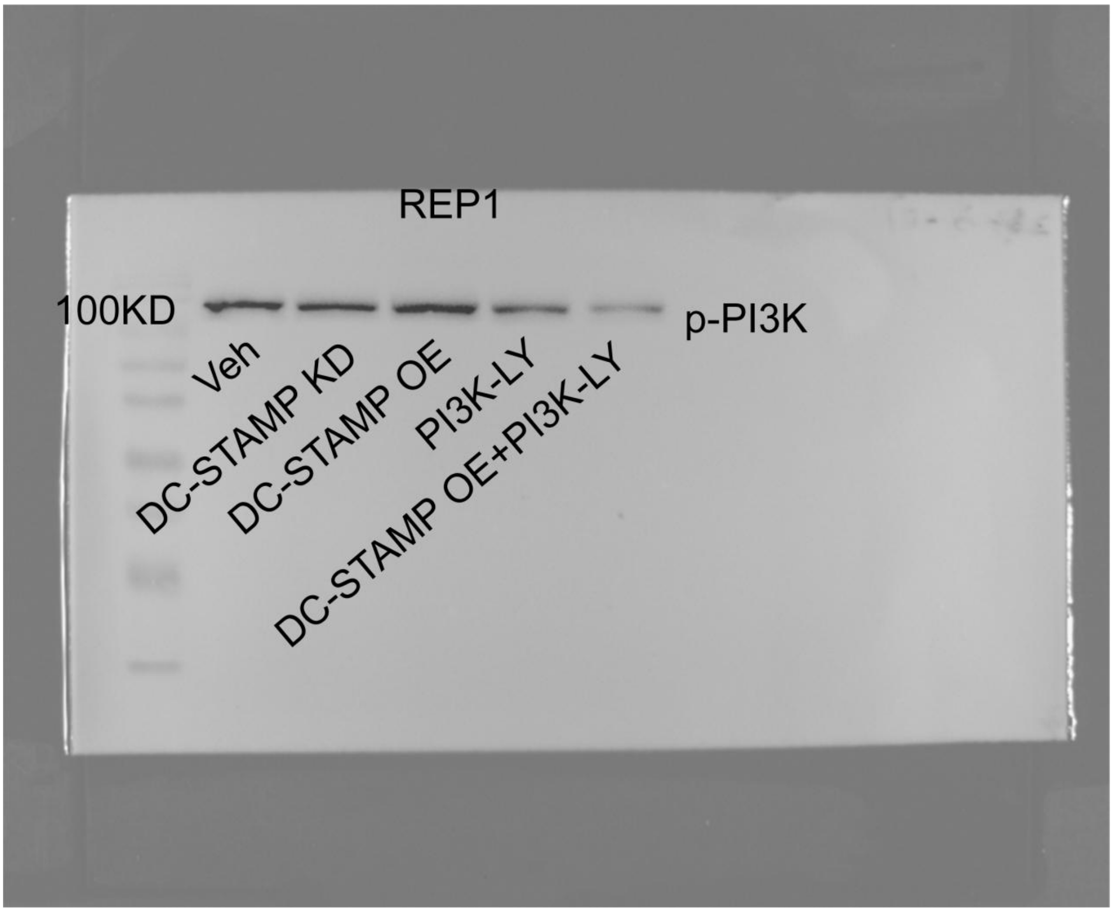
REP1
100KD
p-PI3K
Veh
DC-STAMP KD
DC-STAMP OE
PI3K-LY
DC-STAMP OE+PI3K-LY

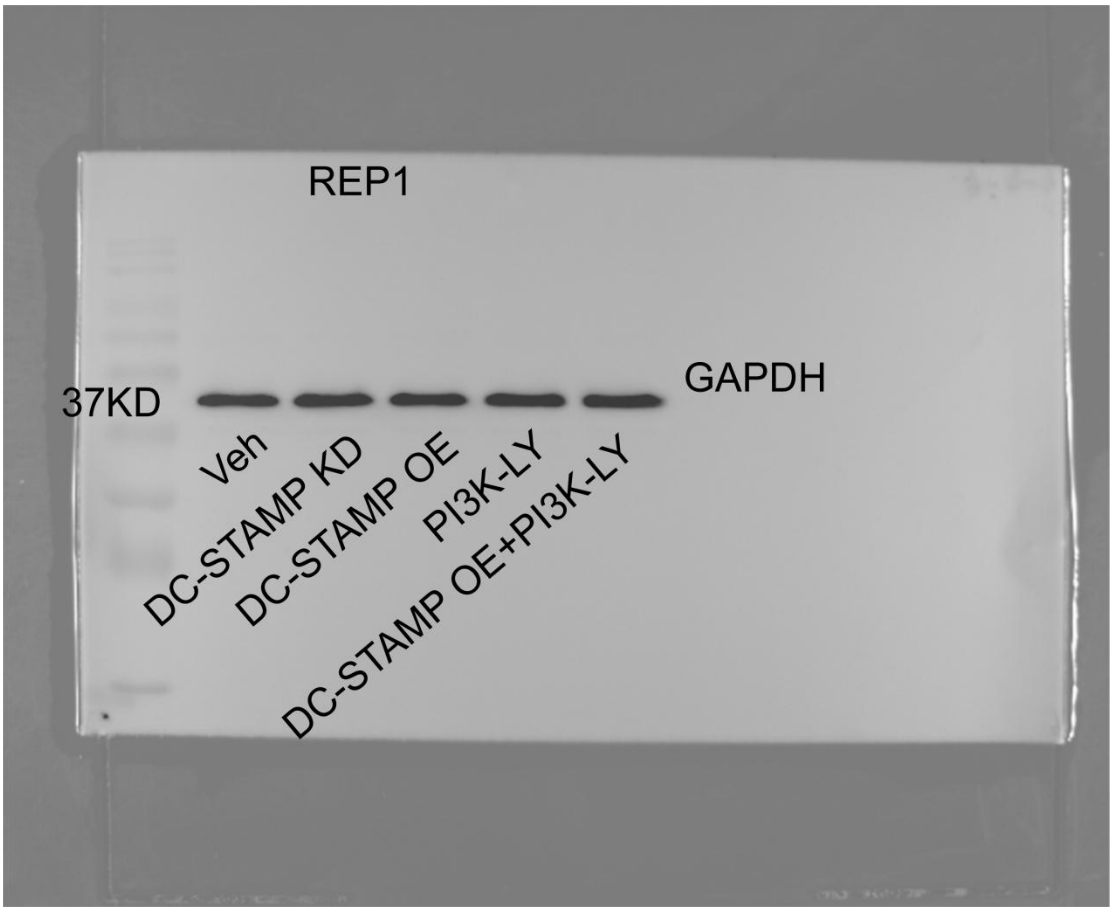
REP1
GAPDH
37KD
Veh
DC-STAMP KD
DC-STAMP OE
PI3K-LY
DC-STAMP OE+PI3K-LY

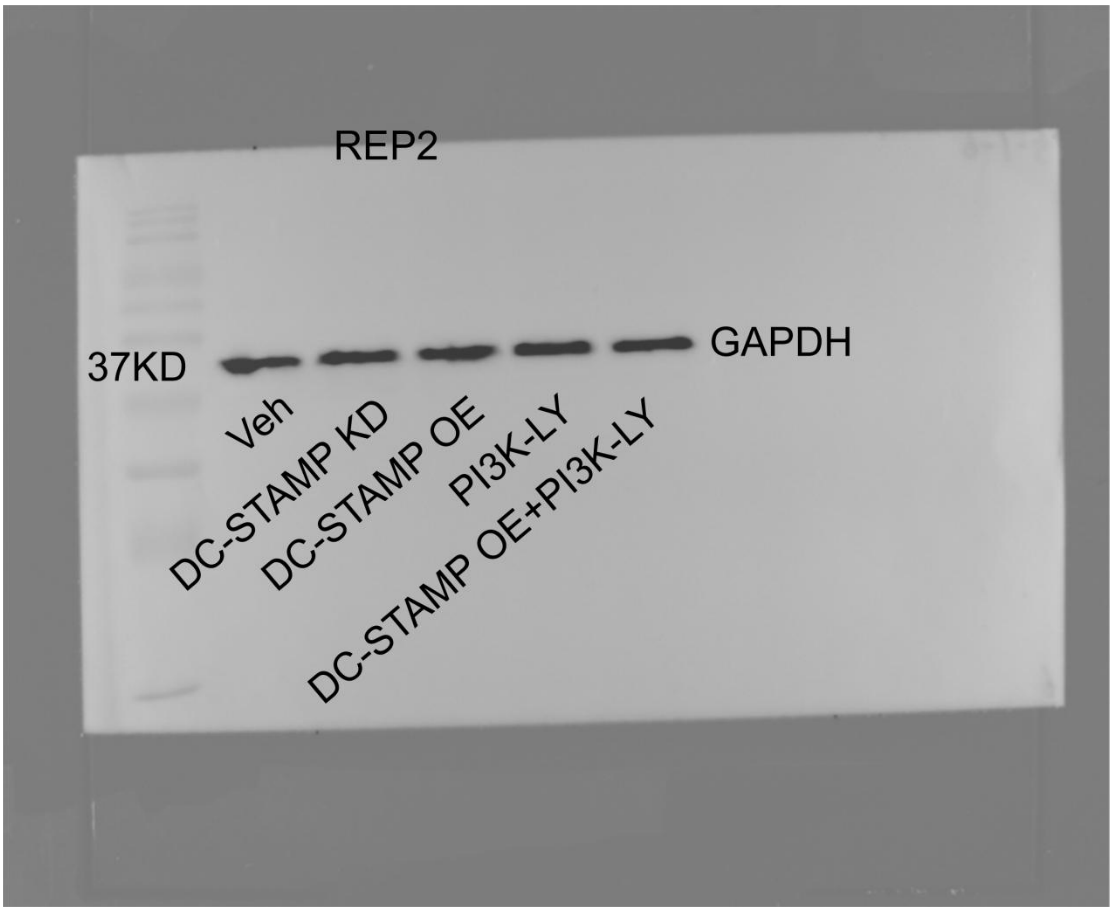
REP2
37KD
GAPDH
Veh
DC-STAMP KD
DC-STAMP OE
PI3K-LY
DC-STAMP OE+PI3K-LY

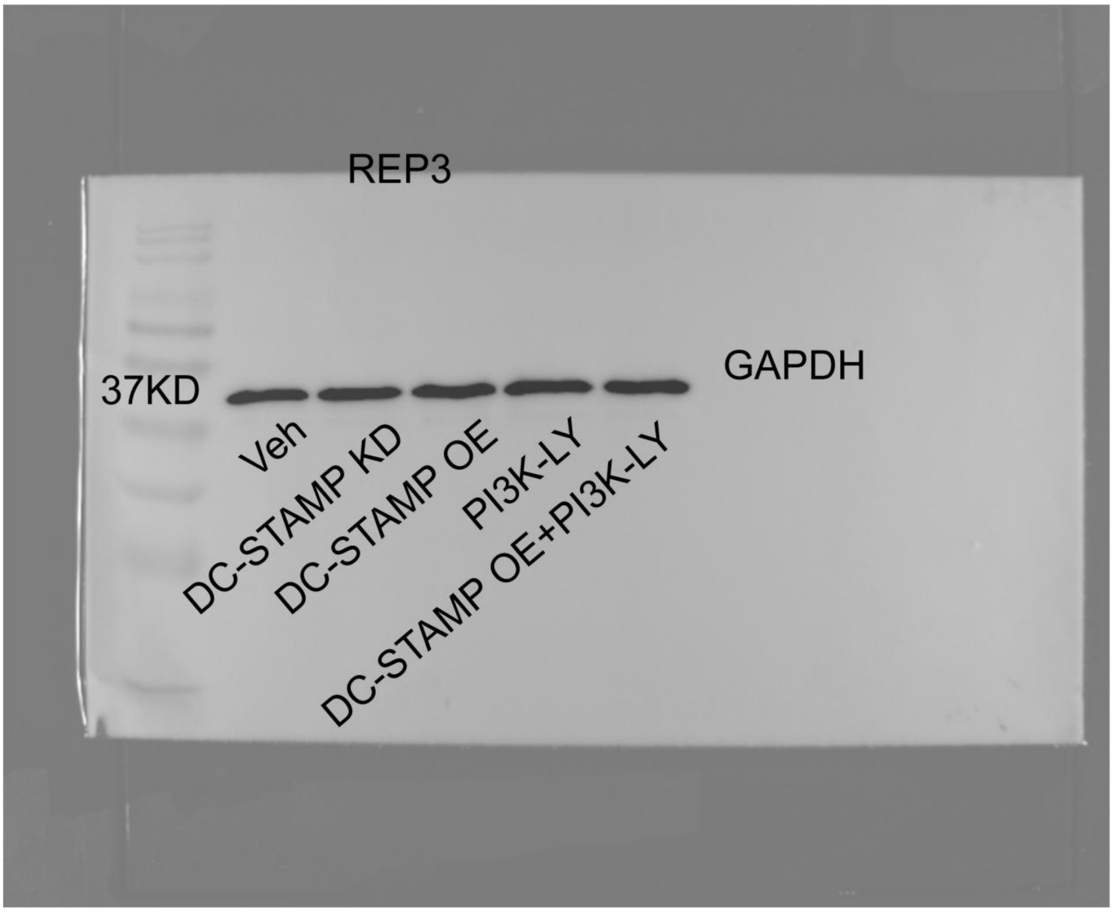
REP3
37KD
GAPDH
Veh
DC-STAMP KD
DC-STAMP OE
PI3K-LY
DC-STAMP OE+PI3K-LY
